# Update of the *Culicoides* (Diptera: Ceratopogonidae) species checklist from Algeria with 10 new records

**DOI:** 10.1186/s13071-020-04335-4

**Published:** 2020-09-10

**Authors:** Mounira Belkharchouche, Selima Berchi, Bruno Mathieu, Ignace Rakotoarivony, Maxime Duhayon, Thierry Baldet, Thomas Balenghien

**Affiliations:** 1 Ecole Nationale Supérieure de Biotechnologie, Taoufik Khaznadar, nouveau pôle universitaire Ali Mendjeli, B.P. E66, 25100 Constantine, Algérie; 2 Faculté des Sciences de la Nature et de la Vie, Université Ibn Khaldoun, B.P.75 Zaaroura, Tiaret, 1400 Algérie; 3 Laboratoire de Biosystématique et Ecologie des Arthropodes, Faculté des Sciences de la Nature et de la Vie, Département de Biologie Animale, Université Frères Mentouri, Constantine 1, 2500 Algérie; 4 CIRAD, UMR ASTRE, 34398 Montpellier, France; 5 Institut de Parasitologie et de Pathologies Tropicales de Strasbourg (IPPTS), UR 7292, 3 Rue Koeberlé, 67000 Strasbourg, France; 6 ASTRE, University of Montpellier, CIRAD, INRAE, Montpellier, France; 7 CIRAD, UMR ASTRE, 97491 Sainte-Clotilde, La Réunion France; 8 CIRAD, UMR ASTRE, 10101 Rabat, Morocco; 9 Institut Agronomique et Vétérinaire Hassan II, Unité Microbiologie, Immunologie et Maladies Contagieuses, 10100 Rabat, Morocco

**Keywords:** Ceratopogonidae, *Culicoides*, New records, Bluetongue, Algeria

## Abstract

**Background:**

The *Culicoides* fauna of Algeria has been historically investigated, leading to the description of many new species by Kieffer in the 1920s, Clastrier in the 1950s or Callot in the 1960s and to a comprehensive inventory by Szadziewski in the 1980s. The emergence of bluetongue in the late 1990s enhanced *Culicoides* collections made in the country over the last two decades, but information remained mostly unpublished. The aim of this study is therefore to provide a comprehensive and updated checklist of *Culicoides* biting midge species in Algeria.

**Methods:**

The literature (published and grey, in French and in English) from 1920 to date on *Culicoides* collections in Algeria was collected and analyzed in the light of the current taxonomic and systematic knowledge and methods. Fresh *Culicoides* material was also analyzed using light/suction trap collections carried out from November 2015 to September 2018 in nine localities of the ‘wilayah’ of Tiaret (northwestern Algeria). Slide mounted specimens were identified morphologically using the interactive identification key IIKC and original descriptions. Specimens were then compared with non-type material originating from different countries and partly with type material.

**Results:**

A total of 13,709 *Culicoides*, belonging to at least 36 species within 10 subgenera, were examined leading to 10 new records in Algeria, including *C. chiopterus*, *C. dewulfi*, *C. navaiae*, *C. grisescens*, *C. paradoxalis*, *C. shaklawensis*, *C. simulator*, *C. univittatus*, *C. achrayi* and *C. picturatus*. These new records and all previous records provided by the literature review were discussed.

**Conclusions:**

We propose a *Culicoides* checklist for the Algerian fauna of 59 valid species, including species mainly with a large Palaearctic distribution and a specific Mediterranean distribution, and only a few species from the Afrotropical region. Among them, several species, mainly of the subgenera *Avaritia* and *Culicoides*, are confirmed or probable vectors of arboviruses important in animal health.
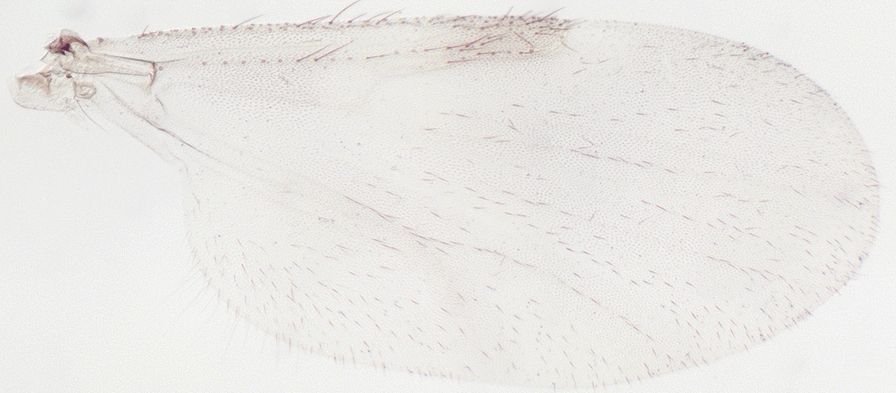

## Background

Biting midges of the genus *Culicoides* Latreille (Diptera: Ceratopogonidae) are small hematophagous dipterans, from 1 to 4 mm long [1]. *Culicoides* are biological vectors involved in the transmission of many arboviruses that affect humans, such as Oropouche virus, ruminants, such as bluetongue virus (BTV) and Schmallenberg virus (SBV), and equids, such as African horse sickness virus (AHSV) [2], but also several parasites, mainly nematodes that can infect humans or animals [3]. Currently, the 1368 valid species described worldwide are placed in 32 subgenera and 38 species groups (not placed to a subgenus), whereas 13% of the world fauna are not placed in any subgenus or group [4].

The worldwide *Culicoides* fauna has been diversely investigated by a number of authors. In the western Palaearctic region, several monographs and catalogues are still considered as a reference for the *Culicoides* fauna [5–7], whereas an interactive identification key has been developed for *Culicoides* females [8]. The Afrotropical fauna has benefited from various contributions, including those of Khamala & Kettle [9] for eastern Africa and Glick [10] for Kenya in particular. Northern Africa is located in the southwestern part of the Palaearctic region and separated from the Afrotropical region by the Sahara Desert. The fauna of this region is composed mainly by species belonging to the Palaearctic fauna, along with some of the Afrotropical fauna.

In the Maghreb, *Culicoides* inventories have been carried out since the beginning of the 20th century, firstly with taxonomic interest enhanced in the 1960s after the emergence of AHSV in Morocco in 1965 [11–14]. In this country, additional ecological studies were carried out after the 1989–1991 AHSV outbreaks [15, 16]. Inventories of *Culicoides* were then re-launched in the Maghreb after the BTV emergence in the Mediterranean basin in the 2000s and the implementation of national-scale entomological surveys [17, 18]. Currently, 54 species of *Culicoides* have been recorded in Morocco [19] and 35 in Tunisia [20]. In Algeria, studies on *Culicoides* have been particularly intense in the first half of the 20th century, with investigations carried out during the 1920s [21–23], the 1930s [24], and the 1950s [25, 26], but also after the 1965 ASHV outbreaks [27]. Altogether, these authors reported the presence of 21 species, including the following 11 still valid species described from Algeria: *C. sergenti* Kieffer; *C. foleyi* Kieffer; *C. nudipennis* Kieffer; *C. parroti* Kieffer; *C. saevus* Kieffer; *C. sahariensis* Kieffer; *C. algeriensis* Clastrier; *C. begueti* Clastrier; *C. cataneii* Clastrier; *C. semimaculatus* Clastrier; and *C. marcleti* Callot, Kremer & Basset. After collections in different regions of Algeria carried out in the early 1980s, Szadziewski [28] reported 30 species, including 19 new records for the Algerian fauna, increasing the number of known species in Algeria to 40. This inventory encompassed several species known as confirmed or probable BTV vectors, such as *C. imicola* Kieffer, *C. obsoletus* Meigen, *C. scoticus* Downes & Kettle within the subgenus *Avaritia* and *C. newsteadi* Austen, *C. pulicaris* (Linnaeus) and *C. punctatus* (Meigen) within the subgenus *Culicoides* [29–33].

From 2000 to 2011, three BTV serotypes were reported in Algeria, leading to 297 outbreaks in 2000 (BTV-2), 263 in 2006 (BTV-1) and six in 2011 (BTV-4) [34, 35]. A few years after these different episodes of BTV transmission, limited-scale surveys for *Culicoides* were carried out in northern and southern Algeria [36–38], before nationwide entomological surveillance was implemented in 2007 by the National Institute of Veterinary Medicine [39, 40]. Moreover, extensive collections have been carried out in the eastern part of Algeria [41, 42]. Altogether, these recent studies increased the total number of species recorded in Algeria to 52 by identifying 12 new species for the Algerian fauna.

In this paper, we aimed to produce a comprehensive checklist of *Culicoides* species in Algeria, including a list of confirmed or probable BTV and AHSV vectors. For this purpose, we compiled and discussed the existing known information from published and from grey literature and examined new material collected using UV light/suction traps carried out from November 2015 to September 2018 in the ‘wilayah’ of Tiaret (northwestern Algeria).

## Methods

Published papers (in French and English) related to *Culicoides* collections in Algeria were collated following searches of classical bibliographic databases (PubMed® and Google Scholar) and on institutional open access repositories (Agritrop from Cirad and Horizon from IRD). The unpublished ‘grey’ literature (field reports and theses) was collated from the two latter databases and from personal databases of authors, but also by contacting authors of the publications and the teams working on *Culicoides* in Algeria. The literature from 1920 to date was analyzed in light of the current taxonomic and systematic knowledge and methods.

We examined *Culicoides* individuals collected using UV light/suction traps, from November 2015 to September 2018, from nine localities in the ‘wilayah’ of Tiaret. The collected *Culicoides* were transferred to 70% ethanol for preservation. The study area is located in the western part of the ‘Hauts Plateaux’ (Highlands or High plateaus), a steppe-like region between the Tell and Saharan Atlas ranges in northwestern Algeria. It is dominated by a continental and semi-arid climate, characterized by very dry summers (300–500 mm per year, with a drought of 6 to 8 months) and cold winters (average annual temperature ranging from 13 to 17 °C) [43]. Mainly dedicated to pastoral activities, the Tiaret region encompasses forested areas composed by cork and holm oaks, Aleppo pine and cypresses.

From the material available, we selected a series of specimens that exhibited a variety of wing patterns, and also those with plain wings. Before identification, the specimens were dissected and slide mounted following the Wirth & Martson [44] procedure. Slide mounted specimens were therefore identified morphologically to the species level using the interactive identification key IIKC of Mathieu et al. [8] and original descriptions. The specimens were then compared with non-type material from different countries and partly with type material available in the collection of the Institut de parasitologie et de pathologie tropicale de Strasbourg, France. To illustrate the new recordings, the wings were photographed using a Zeiss Standard 25 microscope (Zeiss, Oberkochen, Germany) equipped with a Nikon DS-Fi3 camera and Nikon NIS elements v4.6 software (Nikon corporation, Tokyo, Japan). The morphological terminology follows that of Mathieu et al. [8] and Ramilo et al. [45]. With the exception of the species composition of the subgenus *Sensiculicoides*, which follows Szadziewski et al. [46], the subgeneric placement of species other than the latter subgenus and distribution follow Szadziewski et al. [47].

To confirm morphological identification, a small number of specimens were sequenced for the cytochrome *c* oxidase subunit 1 (*cox*1) barcode region following the same procedure detailed in Bourquia et al. [19].

## Results

A total of 13,709 *Culicoides*, belonging to at least 36 species within 10 subgenera, were collected in nine localities of the Tiaret region from November 2015 to September 2018. The results will be analyzed in detail in another publication. Here, we report the 10 new records from Algeria, with special emphasis on *C. navaiae*. The list of the examined material is presented in Table 1.

**Table 1:** List of the material examined to produce 10 new records for the Algerian fauna

Species	Type of material	Country	Locality	Coordinates	Date	Material examined
*Culicoides* (*Avaritia*) *chiopterus* (Meigen)	Non-type material	Algeria	Ain El Deheb	34°51′N, 1°29′E	23–24 Mar 2018	1 female
		France	Andevanne	49°23′N, 5°4′E	17–18 Oct 2006	1 female
			Caro	43°8′N, 1°14′W	23–24 Oct 2006	1 female
			Nuillé sur Vicoin	47°57′N, 0°45′W	4–5 Jul 2011	1 female
			Orbey	48°7′N, 7°7′E	25–26 May 2009	5 females
			Pontivy	48°4′N, 2°58′W	18–19 Jan 2012	5 females
			Schwobsheim	48°13′N, 7°34′E	18–19 Apr 2011	1 female
*Culicoides* (*Avaritia*) *dewulfi* Goetghebuer	Non-type material	Algeria	Tiaret	35°23′N, 1°21′E	16–19 Sep 2017	1 female
			Bougara	35°29′N, 1°55′E	13–15 Nov 2016	1 female
			Sougueur	35°09′N, 1°31′E	13–16 May 2016	2 females
			Ain El Deheb	34°51′N, 1°29′E	25–27 Jul 2017	3 females
			Ain El Deheb	34°51′N, 1°29′E	1–2 Jun 2018	1 female
			Hammadia	35°26′N, 1°53′E	2–5 Jun 2016	1 female
			Takhmaret	35°06′N, 0°42′E	12–15 Sep 2017	1 female
			Takhmaret	35°06′N, 0°42′E	13–14 Sep 2018	3 females
		France	Caro	43°84N, 1°14′W	29–30 May 2006	2 females
			Cuguen	48°26′N, 1°39′W	14–15 Nov 2011	1 female
			Lazer	44°21′N, 5°51′E	18–19 Apr 2011	2 females
*Culicoides* (*Beltranmyia*) *navaiae* Lane	Non-type material	Algeria	Tiaret	35°23′N, 1°21′E	3–6 Jun 2017	2 females
			Tiaret	35°23′N, 1°21′E	28–30 Sep 2017	1 male, 2 females
			Tiaret	35°23′N, 1°21′E	21–22 Sep 2018	2 females
			Bougara	35°29′N, 1°55′E	9–12 May 2016	3 females
			Bougara	35°29′N, 1°55′E	10–13 Nov 2016	3 females
			Bougara	35°29′N, 1°55′E	15–19 Apr 2017	5 females
			Bougara	35°29′N, 1°55′E	29–30 Jun 2018	1 female
			Sougueur	35°09′N, 1°31′E	3–6 Feb 2016	11 females
			Sougueur	35°09′N, 1°31′E	23–26 Jun 2017	4 females
			Sougueur	35°09′N, 1°31′E	14–15 Sep 2018	2 females
			Ain El Deheb	34°51′N, 1°29′E	2–5 Apr 2016	11 females
			Ain El Deheb	34°51′N, 1°29′E	24–27 Apr 2017	2 females
			Ain El Deheb	34°51′N, 1°29′E	21–22 Sep 2018	27 females
			Ksar Chellala	35°15′N, 2°18′E	13–16 May 2016	20 females
			Ksar Chellala	35°15′N, 2°18′E	1–4 Nov 2017	18 females
			Ksar Chellala	35°15′N, 2°18′E	21–22 Sep 2018	18 females
			Hammadia	35°26′N, 1°53′E	16–19 Jun 2016	12 females
			Hammadia	35°26′N, 1°53′E	1–4 Jul 2017	18 females
			Hammadia	35°26′N, 1°53′E	7–8 Sep 2018	9 females
			Rahouia	35°29′N, 1°03′E	23–26 Jun 2017	22 females
			Rahouia	35°29′N, 1°03′E	9–12 Jul 2017	11 females
			Rahouia	35°29′N, 1°03′E	21–22 Sep 2018	18 females
			Machraa S’fa	35°22′N, 1°03′E	3–6 Jun 2017	12 females
			Machraa S’fa	35°22′N, 1°03′E	23–26 Jun 2017	23 females
			Machraa S’fa	35°22′N, 1°03′E	13–14 Jul 2018	7 females
			Takhmaret	35°06′N, 0°42′E	5–8 Jul 2016	8 females
			Takhmaret	35°06′N, 0°42′E	29 Jul–1 Aug 2017	6 females
			Takhmaret	35°06′N, 0°42′E	8–9 Jun 2018	9 females
		Egypt	Sinaï, Khirba	31°1′N, 32°53′E	25 Apr 1979	10 females
			Sinaï, Khirba	31°1′N, 32°53′E	24 Apr 1979	1 male
*Culicoides* (*Culicoides*) *grisescens* Edwards	Non-type material	Algeria	Sougueur	35°09′N, 1°31′E	5–7 Apr 2017	10 females
			Sougueur	35°09′N, 1°31′E	26–29 May 2017	13 females
			Sougueur	35°09′N, 1°31′E	3–6 Jun 2017	5 females
			Sougueur	35°09′N, 1°31′E	17–20 Jul 2017	13 females
			Sougueur	35°09′N, 1°31′E	25–28 Jul 2017	11 females
			Sougueur	35°09′N, 1°31′E	20–23 Sep 2017	15 females
			Sougueur	35°09′N, 1°31′E	1–3 Oct 2017	12 females
			Sougueur	35°09′N, 1°31′E	1–4 Jul 2018	9 females
			Sougueur	35°09′N, 1°31′E	7–8 Sep 2018	9 females
			Ain El Deheb	34°51′N, 1°29′E	29 Nov –5 Dec 2015	2 females
			Ain El Deheb	34°51′N, 1°29′E	15–17 Sep 2016	15 females
			Ain El Deheb	34°51′N, 1°29′E	5–7 Apr 2017	9 females
			Ain El Deheb	34°51′N, 1°29′E	28–29 Sep 2018	11 females
			Ksar Chellala	35°15′N, 2°18′E	26–29 May 2017	7 females
			Ksar Chellala	35°15′N, 2°18′E	9–12 Nov 2017	6 females
			Ksar Chellala	35°15′N, 2°18′E	21–22 Sep 2018	16 females
			Hammadia	35°26′N, 1°53′E	15–17 Sep 2016	8 females
			Hammadia	35°26′N, 1°53′E	28–30 Sep 2017	7 females
			Hammadia	35°26′N, 1°53′E	1–3 Oct 2017	11 females
			Hammadia	35°26′N, 1°53′E	28–29 Sep 2018	12 females
			Rahouia	35°29′N, 1°03′E	24–27 Jun 2016	5 females
			Rahouia	35°29′N, 1°03′E	12–15 Sep 2017	8 females
			Rahouia	35°29′N, 1°03′E	21–22 Sep 2018	7 females
			Machraa S’fa	35°22′N, 1°03′E	8–10 Sep 2016	9 females
			Machraa S’fa	35°22′N, 1°03′E	26–29 May 2017	9 females
			Machraa S’fa	35°22′N, 1°03′E	15–16 Jun 2018	7 females
			Takhmaret	35°06′N, 0°42′E	20–23 Jun 2016	19 females
			Takhmaret	35°06′N, 0°42′E	5–7 Apr 2017	10 females
			Takhmaret	35°06′N, 0°42′E	28–29 Sep 2018	12 females
		France	Brognon	49°55′N, 4°18′E	2è–28 Jun 2007	1 female
			Marcoux	44°7′N, 6°17′E	22–23 Jul 2009	4 females
*Culicoides* (*Culicoides*) *paradoxalis* Ramilo & Delécolle	Type material	Algeria, France, Corsica	Ain El Deheb	34°51′N, 1°29′E	27–28 Apr 2018	1 female
			Pietra Corbara	42°50′N, 9°26′E	3–4 Jun 2003	1 female (holotype)
			Pietra Corbara	42°50′N, 9°26′E	3–4 Jun 2003	2 female
			Pietra Corbara	42°50′N, 9°26′E	6–7 Jul 2004	1 female
			Porto Vecchio	41°35′N, 9°15′E	22–23 Sep 2005	2 females
			Sarrola Carcopino	42°0′N, 8°50′E	26–27 Jun 2006	1 female
			Sartene	41°38′N, 8°57′E	27–28 Sep 2005	1 female
			Sartene	41°38′N, 8°57′E	12–13 Jun 2002	1 female
			Sartene	41°38′N, 8°57′E	20–21 Jun 2002	1 female
*Culicoides* (*Sensiculicoides*) *shaklawensis* Khalaf	Non-type material	Algeria	Tiaret	35°23′N, 1°21′E	9–14 Mar 2016	1 female
			Tiaret	35°23′N, 1°21’E	15–16 Jun 2018	1 female
			Tiaret	35°23′N, 1°21′E	27–28 Jul 2018	1 female
			Bougara	35°29′N, 1°55′E	29–31 Oct 2016	1 female
		France	Calvi	42°32′N, 8°45′E	23–24 Jun 2003	1 female
			Marcoux	44°7′N, 6°17′E	22–23 Jul 2009	1 female
			Moltifao	42°28′N, 9°7′E	5–6 Aug 2003	1 female
*Culicoides* (*Sensiculicoides*) *simulator* Edwards	Non-type material	Algeria	Tiaret	35°23′N, 1°21′E	1–2 Jun 2018	1 female
		France	Aleria	42°6′N, 9°29′E	8–9 Jun 2004	1 female
			La Chapelle d’Andaine	48°31′N, 0°28′W	19–20 Jun 2012	1 female
			Marcoux	44°7′N, 6°17′E	22–23 Jul 2009	1 female
			Remilly Aillicourt	49°39′N, 4°58′E	24–25 May 2007	1 female
*Culicoides* (*Sensiculicoides*) *univittatus* Vimmer	Non-type material	Algeria	Tiaret	35°23′N, 1°21′E	10–12 Dec 2016	1 male, 1 female
			Tiaret	35°23′N, 1°21′E	15–17 Mar 2017	4 males, 7 females
			Tiaret	35°23′N, 1°21′E	4–5 May 2018	2 females
		France, Corsica	Figari	41°30′N, 9°5′E	10–11 Apr 2006	1 male, 4 females
			Figari	41°30′N, 9°5′E	14–15 Feb 2011	1 female
			Moltifao	42°28′N, 9°7′E	25–26 Apr 2005	1 female
			San Giuliano	42°17′N, 9°32′E	6–7 Mar 2003	2 males
			San Giuliano	42°17′N, 9°32′E	10–11 Feb 2011	1 female
*Culicoides* (*Silvaticulicoides*) *achrayi* Kettle & Lawson	Non-type material	Algeria	Hammadia	35°26′N, 1°53′E	27–28 Apr 2018	1 female
			Sougueur	35°09′N, 1°31′E	15–17 Oct 2016	1 female
			Sougueur	35°09′N, 1°31′E	5–7 Apr 2017	1 female
		France	Crozon	48°16′N, 4°30′W	13–14 Jun 2011	2 females
			Pontivy	48°4′N, 2°58′W	18–19 Jan 2012	3 females
*Culicoides* (*Silvaticulicoides*) *picturatus* Kremer & Deduit	Non-type material	Algeria	Tiaret	35°23′N, 1°21′E	24–27 Jun 2016	1 female, 1 male
			Tiaret	35°23′N, 1°21′E	10–12 Jul 2016	1 female
			Tiaret	35°23′N, 1°21′E	28–29 Sep 2018	1 female
		France	Humes Jorquenay	47°54′N, 5°18′E	6–7 Jun 2011	1 male
			Lazer	44°21′N, 5°51′E	18–19 Apr 2011	1 female
			Porto Vecchio	41°35′N, 9°15′E	29–30 May 2002	2 males
			Saignon	43°51′N, 5°27′E	6–7 Jun 2011	3 females

**Fig. 1:**
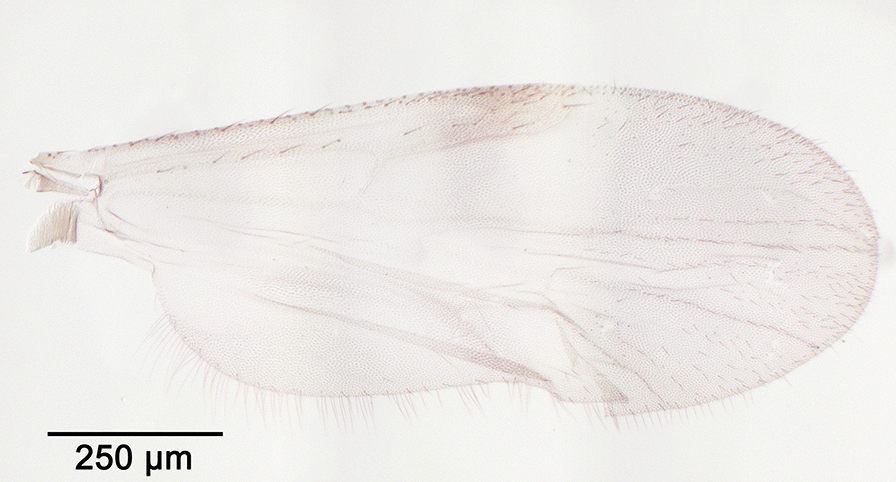
Wing of *Culicoides* (*Avaritia*) *chiopterus* (Meigen)

We report the presence of *Culicoides* (*Avaritia*) *chiopterus* (Meigen) (synonyms *Culicoides amoenus* Winnertz, 1852; *Culicoides similis* Goetghebuer, 1927; *Culicoides dobyi* Callot & Kremer, 1969). Females may be identified using the following criteria. General aspect of the wing light greyish with little contrast as represented in Fig. 1; pale spots poorly defined and barely visible; poststigmatic pale spot covering more than one-third of the 2nd radial cell; absence of pale spots in the distal part of the wing. Hairy eyes with scattered and short interfacetal hairs, and eyes united over a short distance. First abdominal tergite with 1–2 lateral hairs. Two functional spermathecae subequal and one rudimentary. This species is distributed in the Holarctic region, including Austria, Belgium, Czech Republic, Denmark, Estonia, France with Corsica, Germany, Hungary, Ireland, Lithuania, the Near East, the Netherlands, Poland, Romania, Russia, Spain, Slovakia, Switzerland, Turkey, Ukraine [48], the UK and in the Nearctic region. To date, only two species with hairy eyes have been recorded in the Palaearctic region, of which *C. chiopterus* is the only one described from North Africa. We produced a *cox*1 barcode sequence of the *C. chiopterus* female (GenBank: MT782149) to confirm the morphological identification.

**Fig. 2:**
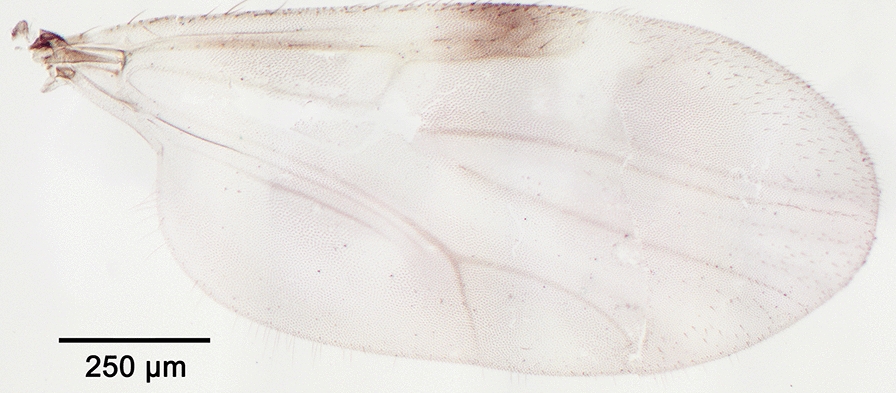
Wing of *Culicoides* (*Avaritia*) *dewulfi* Goetghebuer

We report the presence of *Culicoides* (*Avaritia*) *dewulfi* Goetghebuer (synonym *Culicoides pseudochiopterus* Downes & Kettle, 1952). Females may be identified using the following criteria. General aspect of the wing greyish and well contrasted with the darker part between the 1st and the 2nd radial cells as represented in Fig. 2; pale spots poorly defined; poststigmatic pale spot covering more than one third of the 2nd radial cell; the distal pale spot/area of r3 larger than the pale spot/area in the distal part of m1. Eyes bare and joined over a short distance. First abdominal tergite with 8–12 lateral hairs. Two functional spermathecae unequal and one rudimentary. This Palaearctic species is widely distributed in Europe, including Belgium, Czech Republic, Denmark, Estonia, France with Corsica, Germany, Italy, Poland, Romania, Russia, Slovakia, Spain, Switzerland and the UK [48]. While phylogenetically well separated from species of the Obsoletus group [49], the wing pattern of *C. dewulfi* shares similarities with the latter group. By prior confirmation of slide mounted specimens, the combination of (i) the contrasted wing pattern, (ii) the pale spot/area in the distal part of r3 larger than that of m1 and (iii) the numerous lateral hairs on the first abdominal tergite are good indicators of the species under stereomicroscopy.

**Fig. 3:**
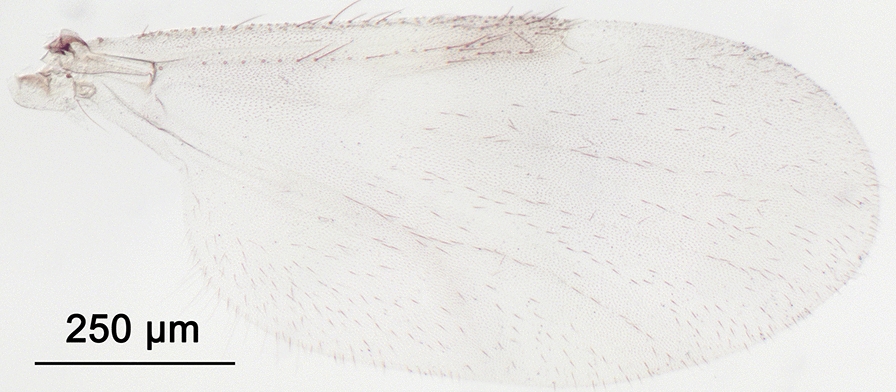
Wing of *Culicoides* (*Beltranmyia*) *navaiae* Lane

**Fig. 4:**
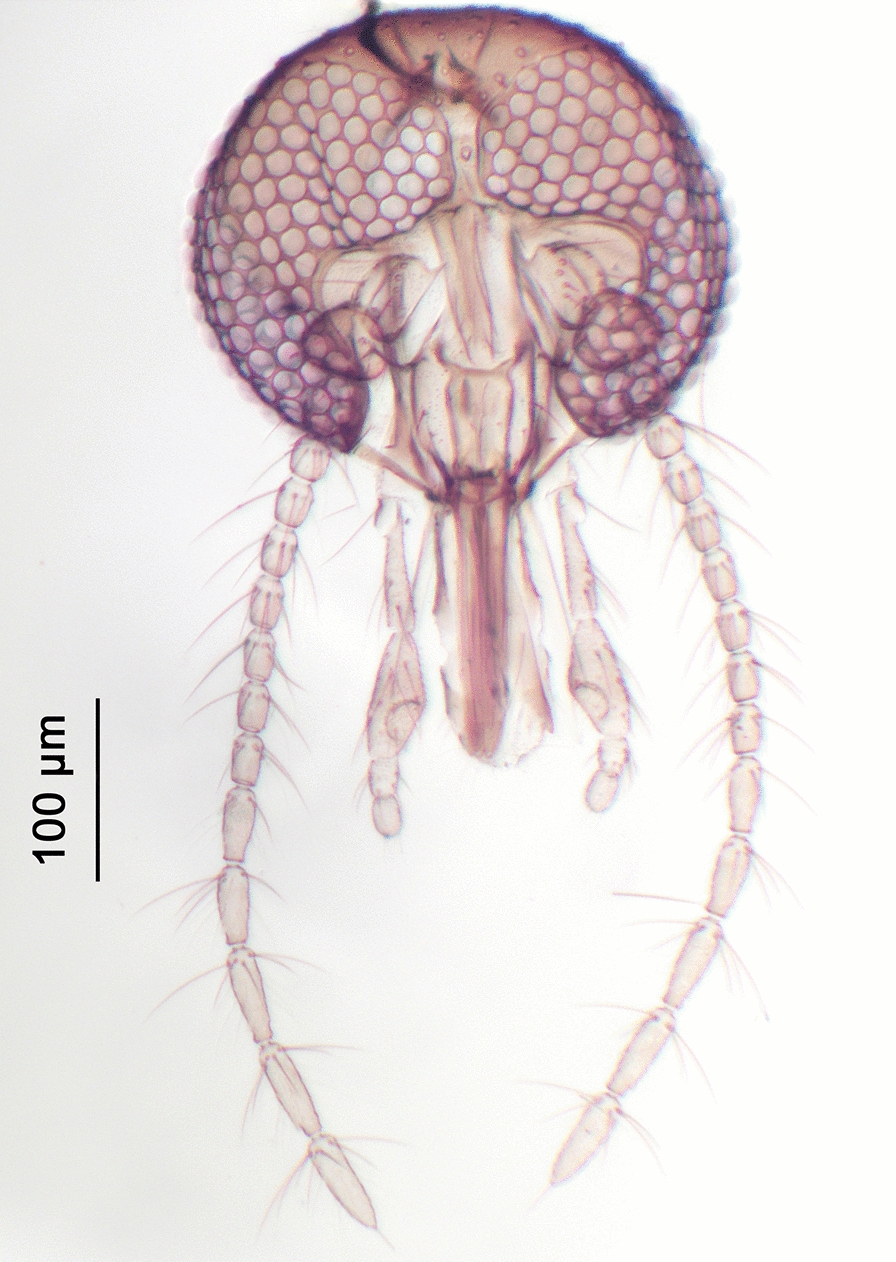
Head of *Culicoides* (*Beltranmyia*) *navaiae* Lane

**Fig. 5:**
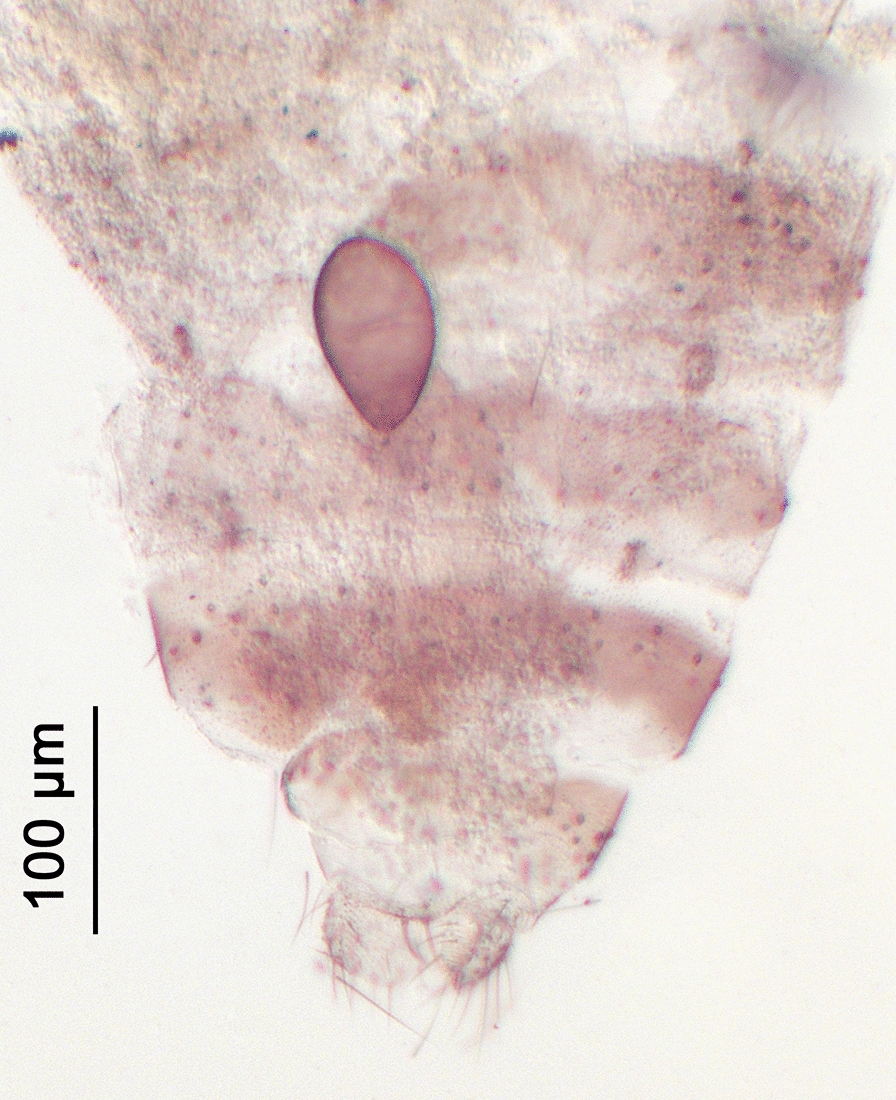
Abdomen (female) of *Culicoides* (*Beltranmyia*) *navaiae* Lane

**Fig. 6:**
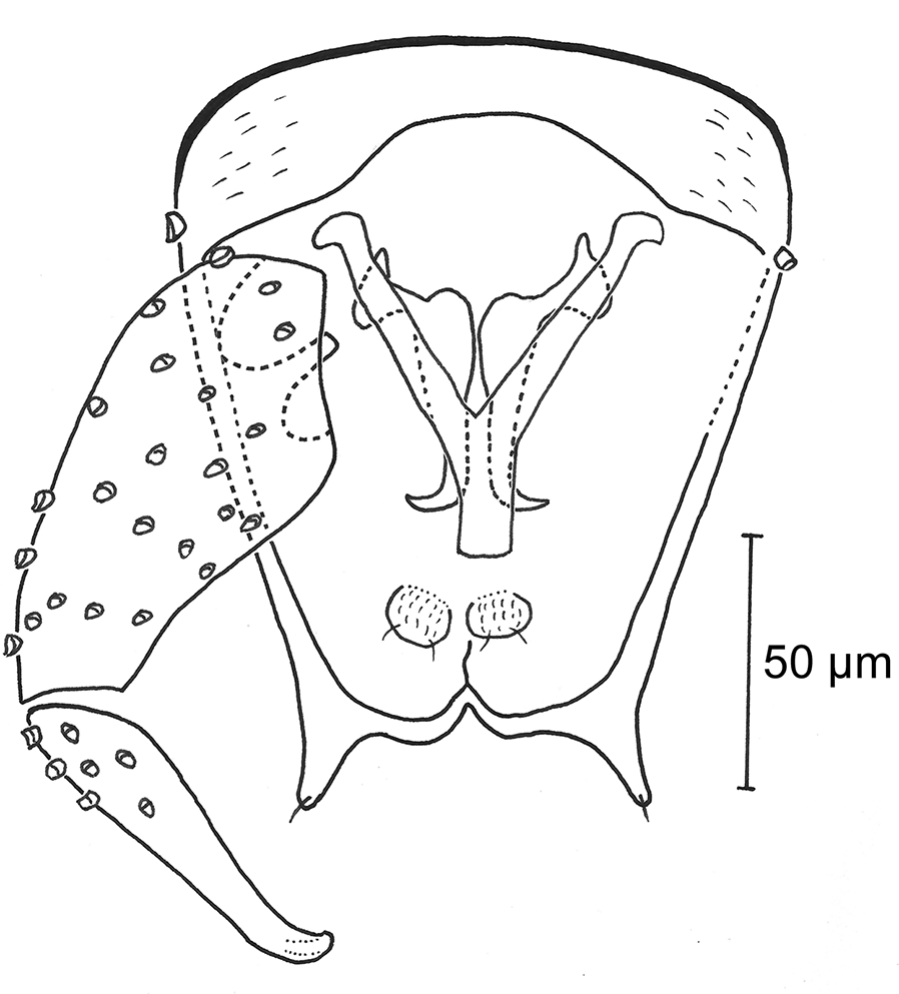
Male genitalia of *Culicoides* (*Beltranmyia*) *navaiae* Lane

We report the presence of *Culicoides* (*Beltranmyia*) *navaiae* Lane. Females may be identified using the following criteria. Wing with a few very faint spots as represented in Fig. 3; no spots in the distal part of r3; absence of macrotrichia in the basal cell. Eyes bare and closely separated (Fig. 4). Sensilla coeloconica present on flagellomeres 1, 9-12. Absence of a postpharyngal armature. Third palpal segment triangular and moderately swollen with single, wide and shallow sensory pit. One functional spermathecae without a pigmented neck (Fig. 5). Males may be identified using the following criteria. Wings pale with fainter spots than in females and barely visible. Ninth tergite broad with a median deep notch, short and divergent apicolateral processes; ninth sternite with bare ventral membrane. Aedeagus with rounded basal arch extending more than two-thirds of the total length, slender and curved-ended basal arms, distal process moderately long (one third of the total length) and blunt-ended (Fig. 6). Parameres separated, swollen at the base and tapering distally to a slender point. This species is reported in the Arabian Peninsula especially in Bahrain, Saudi Arabia, and in the Sinai [50–52]. *Culicoides navaiae* is the only *Beltranmyia* species from the Palaearctic and the Afrotropical regions exhibiting the combination of the following characters: very faint wing pattern close to the plain wing aspect, no spots on the distal part of r3 and sensilla coeloconia distribution on flagellomeres 1, 9-12. The wing pattern of *C. navaiae* may appear similar to the unspotted wing of *C. homochrous* Remm, but the latter has a sensilla coeloconica distribution on flagellomeres 1–2, 7, 9, 11–12 [53]. However, Glukhova [54] reported for *C. homochrous* the sensilla distribution on flagellomeres 1–3, (7), 9–12 and thus the morphological characteristics of this species should be clarified.

**Fig. 7:**
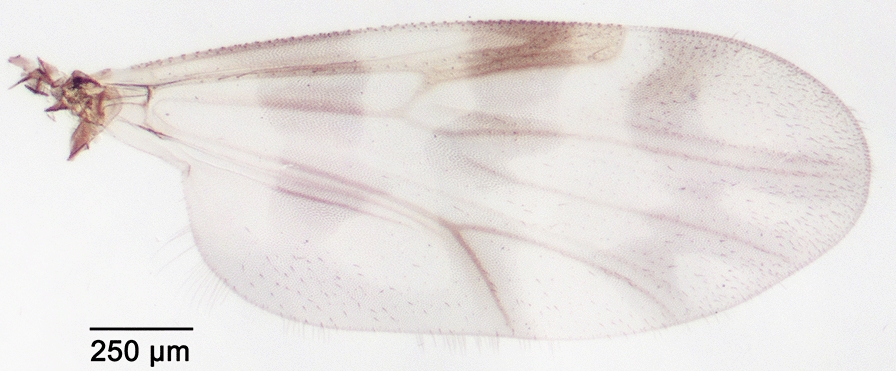
Wing of *Culicoides* (*Culicoides*) *grisescens* Edwards

We report the presence of *Culicoides* (*Culicoides*) *grisescens* Edwards (synonyms *Culicoides remmi* Damian-Georgescu, 1972; *Culicoides arschanicus* Mirzaeva, 1984). Females may be identified using the following criteria. Spotted wing as represented in Fig. 7; cua1 cell pale. Eyes bare, joined over a short distance or separated but connected by a suture. Third palpal segment slender or slightly swollen with multiple and irregular pits. Two functional spermathecae subequal; short pigmented neck and one rudimentary spermathecae. Foreleg and hind leg with spines on first and second tarsomeres; middle leg with spines from first to fourth tarsomeres. This species is distributed in the northern and eastern parts of the Palaearctic region, including Belgium, Denmark [55], Estonia, France, Germany, Poland, Romania, Russia, Slovakia, Switzerland and Ukraine [48]. A phylogenetic study based on ITS2 markers and morphological observations led their authors to suggest that *C. remmi* Damian-Georgescu should be raised from synonymy with *C. grisescens* [56, 57]. We produced a *cox*1 barcode sequence of a *C. grisescens* female (GenBank: MT782148) to confirm the morphological identification.

**Fig. 8:**
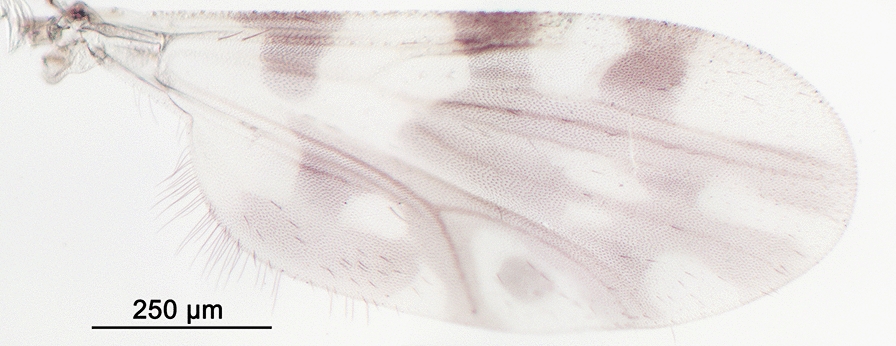
Wing of *Culicoides* (*Culicoides*) *paradoxalis* Ramilo & Delécolle

We report the presence of *Culicoides* (*Culicoides*) *paradoxalis* Ramilo & Delécolle. Females may be identified using the following criteria. Spotted wing as represented in Fig. 8; pale spot in the proximal part of m2 smaller than the one in m1 or absent; poststigmatic pale spot covering half of the 2nd radial cell; cua1 cell centered by a dark spot. Eyes bare and joined over a short distance. Two functional spermathecae subequal and one rudimentary. Middle leg with spines on tarsal segments 1 to 3. This species is distributed in France and Portugal [45]. In recent years, the morphological and molecular diversities within the subgenus *Culicoides* have been studied, leading to the description of several new species [58–60]. Nevertheless, observation of the wing pattern, the pale spot in cell m2 in particular, and the absence of spines on the fourth mid tarsomere should allow the identification of *C. paradoxalis* with confidence.

**Fig. 9:**
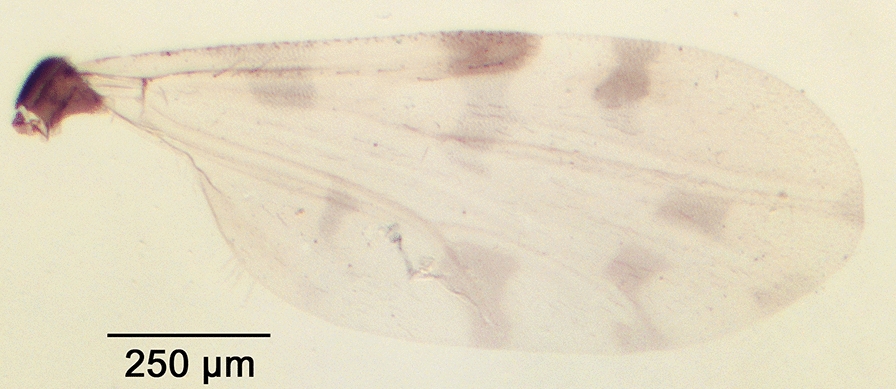
Wing of *Culicoides* (*Sensiculicoides*) *shaklawensis* Khalaf

We report the presence of *Culicoides* (*Sensiculicoides*) *shaklawensis* Khalaf (synonym *Culicoides caspius* Gutsevich, 1959). Females may be identified using the following criteria. Spotted wing as represented in Fig. 9; second radial cell completely dark with the R3 vein slightly covered by the poststigmatic pale spot; most of the veins M1, M2 and CuA1 pale with the exception of the distal tip dark. Eyes bare and separated narrowly. Sensilla coeloconica present on flagellomeres 1, 9–13. Two functional spermathecae unequal and one rudimentary. Middle leg with spines on tarsal segments 1 to 3. Described from Iraq, this species is widespread in the Middle East [61], and reported from Bulgaria, Cyprus [61], Czech Republic, France with Corsica [62], Italy, Morocco, Slovakia, Spain, Tunisia, Turkey and Ukraine. The very characteristic wing pattern of *C. shaklawensis* can be used to identify this species with certainty in the Palaearctic region. In Central Asia, *C. kugitangi* Ataev described in Turkmenistan has a wing pattern similar to that of *C. shaklawanesis* and females can be easily separated from the latter species by the sensilla coeloconica distribution present on all flagellomeres [54].

**Fig. 10:**
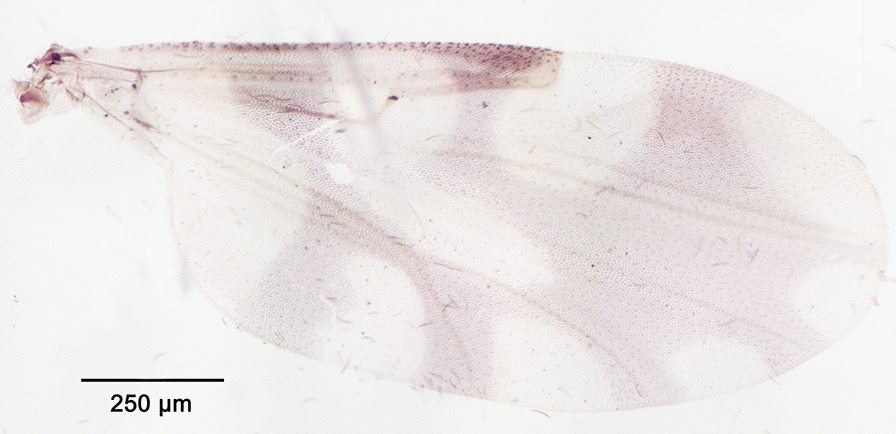
Wing of *Culicoides* (*Sensiculicoides*) *simulator* Edwards

We report the presence of *Culicoides* (*Sensiculicoides*) *simulator* Edwards. Females may be identified using the following criteria. Spotted wing as represented in Fig. 10; poststigmatic pale spot covering one third of the 2nd radial cell; pale spot on the r-m cross vein extending to the proximal part of m2 which layers and crosses the vein M2; distal pale spot in r3 larger than the distal pale spot in m1. Eyes bare and separated narrowly. Sensilla coeloconica present on all flagellomeres 1–13. Two functional spermathecae unequal and one rudimentary; absence of sclerotized ring at the end of the spermathecal duct. Middle leg with spines on tarsal segments 1 to 4. This species is reported from the Balkans [63], Czech Republic, Denmark, Estonia, France, Germany, Hungary, Italy, Lithuania, Morocco, the Netherlands, Poland, Portugal, Russia, Slovakia, Spain, Turkey, Ukraine [64] and the UK. It is the only Palaearctic *Culicoides* with the pale spot on the R-M cross vein extending to the proximal part of m2 cell, which layers and crosses the M2 vein and therefore reaches the proximal part of m1 cell.

**Fig. 11:**
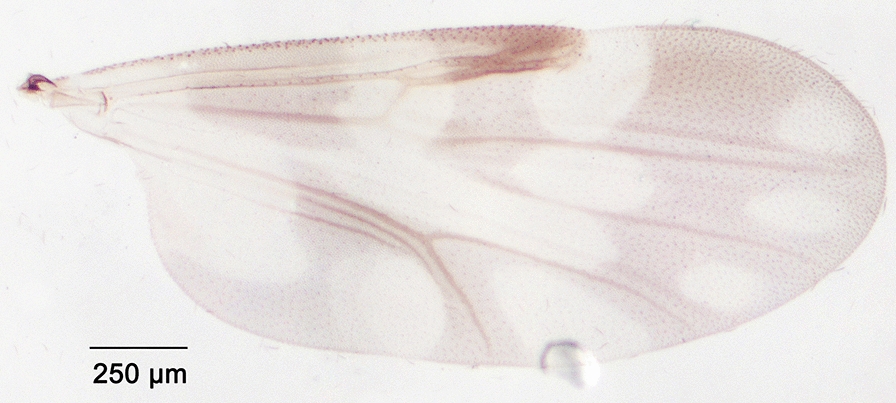
Wing of *Culicoides* (*Sensiculicoides*) *univittatus* Vimmer

We report the presence of *Culicoides* (*Sensiculicoides*) *univittatus* Vimmer (synonym *Culicoides agathensis* Callot, Kremer & Rioux, 1963). Females may be identified using the following criteria. Spotted wing as represented in Fig. 11; second radial cell entirely dark with the R3 vein slightly covered by the poststigmatic pale spot; pale spots on the distal part of r3 and m1 rounded and separated from the wing margin. Eyes bare and separated narrowly. Sensilla coeloconica present on all flagellomeres. Two functional spermathecae unequal and one rudimentary. Male wing similar to female with the usual sexual differences. Aedeagus with rounded basal arch extending about one-third of total length, distal process long (two-thirds of total length), triangular with blunt rounded tip. Parameres separated and tapering to a fine point; ninth sternite with ventral membrane bare or rarely bearing few spicules. This species is reported from Albania, Cyprus [61], France (southern mainland and Corsica), Italy (mainland and Sardinia), Morocco [11, 12], Portugal, Spain, Tunisia [20] and Turkey. This is the only species of *Sensiculicoides* with the pale spots on the distal part of r3 and m1 separated from the wing margin. Very close to *C. pictipennis* (Staeger), the male can be distinguished by the wing pattern and the distal process of the aedeagus, which is triangular for *C. univittatus* and rectangular for *C. pictipennis.*

**Fig. 12:**
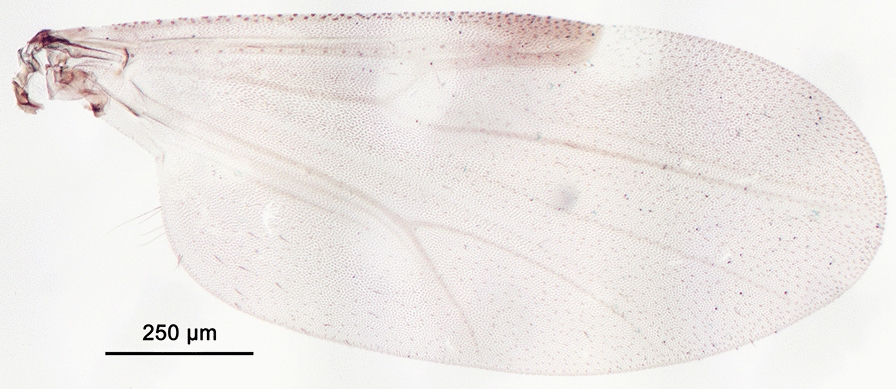
Wing of *Culicoides* (*Silvaticulicoides*) *achrayi* Kettle & Lawson

We report the presence of *Culicoides* (*Silvaticulicoides*) *achrayi* Kettle & Lawson. Females may be identified using the following criteria. Greyish wing with few spots as represented in Fig. 12; second radial cell completely dark with the R3 vein slightly covered by the post-stigmatic pale spot. Eyes bare and separated narrowly. Sensilla coeloconica present on flagellomeres 1, 9-13. Third palpal segment triangular and moderately swollen with multiple irregular sensory pits. Two functional spermathecae subequal with a long, pigmented neck and one rudimentary spermathecae; parallel sclerotized ring at the end of the spermathecal duct. Middle leg with spines on tarsal segments 1 to 4. This species is reported from the Balkans [63], Belgium, Bulgaria, Cyprus, Czech Republic, Denmark, Estonia, France with Corsica, Germany, Hungary, Italy, Ireland, Lithuania, Morocco [65], the Near East, Poland, Portugal, Russia, Slovakia, Switzerland, Ukraine [64] and the UK.

**Fig. 13:**
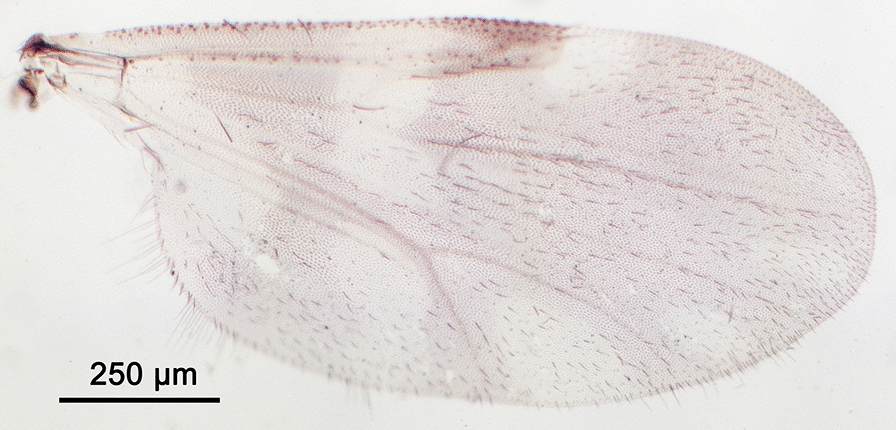
Wing of *Culicoides* (*Silvaticulicoides*) *picturatus* Kremer & Deduit

We report the presence of *Culicoides* (*Silvaticulicoides*) *picturatus* Kremer & Deduit. Females may be identified using the following criteria. Spotted wing as represented in Fig. 13; second radial cell completely dark, small and faint pale spots in the distal part of r3, m1 and m2 cells. Eyes bare and separated narrowly. Sensilla coeloconica present on flagellomeres 1, 9-13. Two functional spermathecae subequal and one rudimentary. Middle leg with spines on tarsal segments 1 to 3. Males may be identified using the following criteria. Wing similar to female with the usual sexual differences. Ninth sternite with broad and deep arch-shape caudomedian excavation, ventral membrane densely spiculate; basistyle with ventral root long and slender. This species was first described in France (Normandy region) by Kremer & Deduit [66], and reported from Corsica, Denmark, Italy (mainland, Sardinia and Sicily), Morocco [11, 12], Portugal, Romania, Slovakia [67], Spain, Switzerland, Turkey, and the UK. *Culicoides alazanicus* Dzhafarov has a wing pattern similar to that of *C. picturatus* but can be easily separated from the latter under stereomiscrocopy by the ratio of the length of flagellomere 9 to that of flagellomere 8 on the female antenna being greater than 2. On the contrary, *C. picturatus* has such a ratio around 1.4 [67].

## Discussion

We report ten new records of *Culicoides* species in Algeria (Table 2) by examining fresh material collected from November 2015 to September 2018 using light/suction traps in nine localities of the Tiaret region (northwestern Algeria). Among these ten species new to Algeria, *C. shaklawensis*, *C. simulator* and *C. picturatus* were already known from Morocco and *C. univittatus* from both Morocco and Tunisia [19, 20].

**Table 2:** *Culicoides* species list of Algeria

Subgenus	Species	Additional records from our data	Proposed checklist	References
*Avaritia* Fox, 1955	*Culicoides chiopterus* Meigen, 1830	×	×	Present study
	*Culicoides dewulfi* Goetghebuer, 1936	×	×	Present study
	*Culicoides imicola* Kieffer, 1913		×	[28, 36, 38–42]
	*Culicoides montanus* Shakirzjanova, 1962		×	[39]
	*Culicoides obsoletus* Meigen, 1818		×	[22, 24, 25, 28, 36, 40–42]^a^
	*Culicoides scoticus* Downes & Kettle, 1952		×	[28, 36, 41, 42]
*Beltranmyia* Vargas, 1953	*Culicoides circumscriptus* Kieffer, 1918		×	[25, 27, 28, 36, 40–42]
	*Culicoides navaiae* Lane, 1983	×	×	Present study
	*Culicoides sphagnumensis* Williams, 1955			[42]
*Culicoides* Latreille, 1809	*Culicoides fagineus* Edwards, 1939		×	[25, 41, 42]
	*Culicoides grisescens* Edwards, 1939	×	×	Present study
	*Culicoides newsteadi* Austen, 1921		×	[28, 36, 40–42]^b^
	*Culicoides paradoxalis* Ramilo & Delécolle, 2013	×	×	Present study
	*Culicoides pulicaris* (Linnaeus, 1758)		×	[28, 41, 42]
	*Culicoides punctatus* (Meigen, 1804)		×	[28, 36, 41, 42]
*Monoculicoides* Khalaf, 1954	*Culicoides parroti* Kieffer, 1922		×	[22, 23, 36]
	*Culicoides puncticollis* (Becker, 1903)		×	[22, 23, 25, 28, 36, 40–42]^c^
*Oecacta* Poey, 1853	*Culicoides azerbajdzhanicus* Dzhafarov, 1962		×	[28, 37]
	*Culicoides corsicus* Kremer, Leberre & Beaucournu-Saguez, 1971		×	[36]
	*Culicoides longipennis* Khalaf, 1957		×	[27, 36]
	*Culicoides marcleti* Callot, Kremer & Basset, 1968		×	[27, 28]
	*Culicoides ravus* de Meillon, 1936		×	[37]
	*Culicoides sahariensis* Kieffer, 1923		×	[23, 25, 28, 36, 40, 42]^d^
	*Culicoides santonicus* Callot, Kremer, Rault & Bach, 1966		×	[28]
	*Culicoides semimaculatus* Clastrier, 1958		×	[26]
	*Culicoides sergenti* Kieffer, 1921		×	[21, 23, 28, 37]^e^
	*Culicoides truncorum* Edwards, 1939			[42]
*Pontoculicoides* Remm, 1968	*Culicoides saevus* Kieffer, 1922		×	[22–24, 28, 36, 41, 42]
	*Culicoides sejfadinei* Dzhafarov, 1958		×	[28]
*Remmia* Glukhova, 1977	*Culicoides kingi* Austen, 1912		×	[28, 37, 40–42]
	*Culicoides schultzei* (Enderlein, 1908)		×	[25, 42]
*Sensiculicoides* Shevchenko, 1977	*Culicoides begueti* Clastrier, 1957		×	[25]
	*Culicoides cataneii* Clastrier, 1957		×	[25, 28, 36, 40]
	*Culicoides clastrieri* Callot, Kremer & Deduit, 1962		×	[39, 41, 42]
	*Culicoides duddingstoni* Kettle & Lawson, 1955		×	[42]
	*Culicoides dzhafarovi* Remm, 1967		×	[28]
	*Culicoides festivipennis* Kieffer, 1914		×	[36]
	*Culicoides gejgelensis* Dzhafarov, 1964		×	[27, 28, 36]
	*Culicoides griseidorsum* Kieffer, 1918		×	[28, 41, 42]
	*Culicoides heteroclitus* Kremer & Callot, 1965		×	[27, 28, 36]
	*Culicoides jumineri* Callot & Kremer, 1969		×	[28, 36, 42]
	*Culicoides jurensis* Callot, Kremer & Deduit, 1962			[42]
	*Culicoides kibunensis* Tokunaga, 1937		×	[27]^f^
	*Culicoides kurensis* Dzhafarov, 1960		×	[36]
	*Culicoides langeroni* Kieffer, 1921		×	[28, 37, 40]
	*Culicoides maritimus* Kieffer, 1924		×	[28, 41, 42]
	*Culicoides odiatus* Austen, 1921		×	[28, 36]
	*Culicoides pictipennis* (Staeger, 1839)		×	[28, 41, 42]
	*Culicoides poperinghensis* Goetghebuer, 1953		×	[28]
	*Culicoides pseudopallidus* Khalaf, 1961		×	[27, 28, 36]
	*Culicoides shaklawensis* Khalaf, 1957	×	×	Present study
	*Culicoides simulator* Edwards, 1939	×	×	Present study
	*Culicoides univittatus* Vimmer, 1932	×	×	Present study
*Silvaticulicoides* Glukhova, 1977	*Culicoides achrayi* Kettle & Lawson, 1955	×	×	Present study
	*Culicoides fascipennis* (Staeger, 1839)		×	[36]
	*Culicoides picturatus* Kremer & Deduit, 1961	×	×	Present study
	*Culicoides subfasciipennis* Kieffer, 1919		×	[28]
*Wirthomyia* Vargas, 1973	*Culicoides faghihi* Navai, 1971		×	[28]
Miscellaneous unplaced species	*Culicoides algeriensis* Clastrier, 1957		×	[25]
	*Culicoides foleyi* Kieffer, 1922		×	[22]
	*Culicoides nudipennis* Kieffer, 1922		×	[22]
	*Culicoides paolae* Boorman, Mellor & Scaramozzino, 1996		×	[36]

^a^ Reported as *Culicoides kabyliensis* n. sp. Kieffer, 1922 in [22]

^b^ Reported as *Culicoides halophilus* Kieffer, 1924 in [40]

^c^ Reported as *Culicoides distigma* n. sp. Kieffer, 1922 and *Culicoides donatieni* n. sp. Kieffer, 1922 in [22]

^d^ Reported as *Culicoides coluzzii* Callot, Kremer & Bailly-Choumara, 1970 in [42]

^e^ Reported as *Culicoides citrinellus* n. sp. Kieffer, 1923 in [23]

^f^ Reported as *Culicoides cubitalis* Edwards, 1939 in [27]

*References*: [21] Kieffer (1921); [22] Kieffer (1922); [23] Kieffer (1923); [24] Goetghebuer (1939); [25] Clastrier (1957); [26] Clastrier (1958); [27] Callot et al. (1968); [28] Szadziewski (1984); [36] Baldet et al. (2003); [37] Baldet & Delécolle (2006); [38] Nolan et al. (2008); [39] Djerbal & Delécolle (2009); [41] Belkharchouche (2014); [42] Kabbout (2017); [40] Berrayah et al. (2020)

We also recorded the presence of *C. chiopterus* (one female) and *C. dewulfi* (13 females). Both species are widespread in Europe, but rare in the Mediterranean area. This is the first record of these species in North Africa, which underlines the need for further investigations to clarify their distribution in Algeria and more widely in the Maghreb. Currently, six species of the subgenus *Avaritia* are reported in Algeria (Table 2).

Among these species, *C. imicola* is a proven BTV and AHSV vector species: it is a livestock and equid biting species; numerous isolations of both viruses have been made from field-collected individuals; and the entire cycle of transmission of both viruses has been experimentally reproduced [68, 69]. *Culicoides imicola* can be considered a probable SBV vector, as the viral genome was recovered from field-collected females [70, 71]. *Culicoides obsoletus*, *C. scoticus*, *C. chiopterus* and *C. dewulfi* are probable BTV and SBV vectors because of their ecological habits, and virus isolation or viral genome detection from field-collected individuals and experimental infections. BTV has been isolated from field-collected females, reported as ‘*C. obsoletus*’ taxon [72–74]; however, it was not clear whether this taxon referred to a species or an assemblage of species. BTV-8 genome has been identified from *C. dewulfi* and *C. chiopterus* field-collected individuals by real-time RT-PCR in the Netherlands [75, 76] and France [77]. BTV-1 genome was detected by real-time RT-PCR in *C. obsoletus/C. scoticus* parous females in the Basque country [78], and in *C. obsoletus* and *C. scoticus* parous females in Sardinia [79]. SBV genome has been identified in field-collected individuals: entire females of *C. obsoletus/C. scoticus* in Italy, the Netherlands and Poland [80–82] and of *C. obsoletus*, *C. scoticus*, *C. dewulfi* and *C. chiopterus* in France [70], and heads of *C. obsoletus*, *C. scoticus*, *C. chiopterus* and *C. dewulfi* in Belgium, Denmark and/or the Netherlands [83–85]. Moreover, *C. obsoletus* and *C. scoticus* from the UK have been experimentally infected with BTV-8 and BTV-9; *C. scoticus* exhibiting higher viral titers [86]. Dissemination efficiency has been estimated to be about 20% for BTV-1 in *C. scoticus* populations in Switzerland [32].

A total of 287 females and 1 male collected from the ‘wilahya’ of Tiaret were identified as *C. grisescens*. This species has been reported to be widely distributed in the northern part of the western Palaearctic region, but has never been recorded in North Africa. This species does not seem an efficient experimental vector for BTV [32]. We reported the presence of *C. paradoxalis* (1 female). This species has recently been described in southeastern France, Corsica and Portugal [45]. This new record confirms the Mediterranean distribution of *C. paradoxalis*. Currently, seven species of the subgenus *Culicoides* are reported in Algeria (Table 1). Among these, *C newsteadi*, *C. pulicaris* and *C. punctatus* are considered possible BTV and SBV vectors, due to their feeding habits and to virus isolation or viral genome detections from field-collected individuals. In Italy, BTV has been isolated from *C. pulicaris* [87]; however, it was not clear whether this taxon referred to a species or an assemblage of species. BTV genome has been detected in field-collected individuals of *C. newsteadi*, *C. pulicaris* and *C. punctatus* in Spain and Italy [30, 33, 78, 79], while SBV genome has been detected in *C. punctatus* in Poland [82] and *C. newsteadi* and *C. pulicaris* in France [70].

We reported the presence of *C. navaiae* (286 females and 1 male), first described in Saudi Arabia: females by Lane [50] and males by Boorman [51]. This species of the subgenus *Beltranmyia* was relatively common in Saudi Arabia [88, 89], reported in the Sinai, Egypt [52], and recorded for the first time in our study in the Maghreb. When studying specimens from Algeria, Szadziewski [28] described a single female under the label *C.* (*Beltranmyia*) sp. indet. aff*. homochrous* because identification remained unsatisfactory. With the exception of the presence of sensilla coeloconica on the flagellomere 2 of the latter unidentified female [28], all characters are in agreement with the descriptions of *C. navaiae* females [50, 52] and with our observations. Consequently, we agreed with Boorman’s remark [51] that the female *C.* (*Beltranmyia*) sp. indet. aff*. homochrous* from Szadziewski [28] referred probably to *C. navaiae.*

We collected three females identified as *C. achrayi*. This species has a wide distribution in the western Palaearctic region, including Mediterranean areas. This species has been collected in 2017 in Morocco as the first record in North Africa [65].

Kabbout [42] reported the first records of *C. jurensis*, *C. sphagnumensis* and *C. truncorum* from Algeria. As these species are rare and exhibit a non-Mediterranean Palaearctic distribution, we suggest not validating these species in the *Culicoides* checklist of Algeria until confirmation of identification by experts and molecular confirmation (Table 2).

The proposed checklist includes 59 *Culicoides* species in Algeria, while 54 species are recorded in Morocco [19] and 35 in Tunisia [20]. The Algerian fauna encompasses:

i. 28 species (47.5% of the species) with a wide and non-Mediterranean Palaearctic distribution: *C. achrayi*, *C. begueti*, *C. chiopterus*, *C. circumscriptus*, *C. clastrieri*, *C. dewulfi*, *C. duddingstoni*, *C. fagineus*, *C. fascipennis*, *C. festivipennis*, *C. foleyi*, *C. grisescens*, *C. kibunensis*, *C. kurensis*, *C. montanus*, *C. navaiae*, *C. nudipennis*, *C. obsoletus*, *C. odiatus*, *C. pictipennis*, *C. picturatus*, *C. pulicaris*, *C. punctatus*, *C. saevus*, *C. scoticus*, *C. shaklawensis*, *C. simulator* and *C. subfasciipennis*;
ii. 28 species (47.5% of the species) with a Mediterranean distribution: *C. algeriensis*, *C. azerbajdzhanicus*, *C. cataneii*, *C. corsicus*, *C. dzhafarovi*, *C. faghihi*, *C. gejgelensis*, *C. griseidorsum*, *C. heteroclitus*, *C. jumineri*, *C. langeroni*, *C. longipennis*, *C. marcleti, C. maritimus*, *C. newsteadi*, *C. paolae*, *C. paradoxalis*, *C. parroti*, *C. poperinghensis*, *C. puncticollis*, *C. pseudopallidus*, *C. ravus*, *C. sahariensis*, *C. santonicus*, *C. sejfadinei*, *C. semimaculatus*, *C. sergenti* and *C. univittatus*;
iii. 3 species (5.0% of the species) with an Afrotropical distribution: *C. imicola*, *C. kingi* and *C. schultzei*.

Among these species, *C. kingi* is considered a probable vector of *Onchocerca gutturosa* affecting cattle in the Sahelian region [90, 91] and a potential vector of epizootic hemorrhagic disease virus [92, 93]. Recently, the detection of BTV genome in the head of *C. circumscriptus* and *C. paoale* in Sardinia has raised the question of the possible involvement of these species in BTV transmission in the Mediterranean basin [33].

## Conclusions

The examination of fresh material resulted in the recording of ten new species for the Algerian fauna, although this material was collected in a small part of the country, namely in the ‘wilahya’ of Tiaret (northwestern Algeria). We have combined this information with existing published and grey literature to produce a comprehensive *Culicoides* checklist for Algeria of 59 species, including potential and probable vectors of arboviruses of veterinary interest. This is a prerequisite for the development of a barcode library and an atlas of diagnostic characters. Both may be useful for further ecological studies, in order to establish risk mapping for *Culicoides*-borne diseases in Algeria.

## Data Availability

The material is available on request and is held at the Institut de Parasitologie et de Pathologie Tropicale de Strasbourg (France) and at the Laboratoire de Biosystématique et Ecologie des Arthropodes, Faculté des Sciences de la Nature et de la Vie, University of Constantine 1 (Algeria).

## Abbreviations

AHSV: African horse sickness virus
BTV: bluetongue virus
SBV: Schmallenberg virus
CIRAD: Centre de Coopération Internationale en Recherche Agronomique pour le Développement
IRD: Institut de Recherche pour le Développement
*cox*1: cytochrome *c* oxidase subunit 1
ITS2: internal transcribed spacer 2
RT-PCR: reverse transcription polymerase chain reaction
UV: ultraviolet
IIKC: interactive identification key for *Culicoides*
cua1: first cubital cell
CuA1: first cubital vein
m1: first median cell
m2: second median cell
M1: first median vein
M2: second median vein
r3: third radial cell
R3: third radial vein
R-M: radial-median (cross vein)

## References

[1] Garros C, Balenghien T. Chapitre 14. Les culicoïdes (Diptera : Ceratopogonidae). In: Duvallet G, Fontenille D, Robert V, editors. Entomologie médicale et vétérinaire. Marseille: IRD Éditions & Versailles: Éditions Quae; 2017.

[2] Purse BV, Carpenter S, Venter GJ, Bellis G, Mullens BA (2015). Bionomics of temperate and tropical *Culicoides* midges: knowledge gaps and consequences for transmission of *Culicoides*-borne viruses. Annu Rev Entomol..

[3] Linley JR (1985). Biting midges (Diptera: Ceratopogonidae) as vectors of nonviral animal pathogens. J Med Entomol..

[4] Borkent A. The subgeneric classification of species of *Culicoides* 2016—thoughts and a warning. 2016. https://www.inhs.illinois.edu/files/5014/6532/8290/CulicoidesSubgenera.pdf. Accessed 3 June 2020.

[5] Campbell JA, Pelham-Clinton EC (1960). Taxonomic review of the British species of *Culicoides* Latreille (Diptera, Ceratopogonidae). Proc R Soc Edinburgh B Biol..

[6] Kremer M (1965). Genre *Culicoides* Latreille.

[7] Delécolle JC. Nouvelle contribution à lʼétude systématique et iconographique des espèces du genre *Culicoides*, (Diptéra: Cératopogonidae) du Nord-Est de la France. PhD thesis, Université Louis Pasteur de Strasbourg I, France; 1985.

[8] Mathieu B, Cêtre-Sossah C, Garros C, Chavernac D, Balenghien T, Carpenter S (2012). Development and validation of IIKC: an interactive identification key for *Culicoides* (Diptera: Ceratopogonidae) females from the western Palaearctic region. Parasit Vectors..

[9] Khamala CM, Kettle DS (1971). The *Culicoides* Latreille (Diptera: Ceratopogonidae) of East Africa. Trans R Ent Soc Lond..

[10] Glick JI (1990). *Culicoides* biting midges (Diptera: Ceratopogonidae) of Kenya. J Med Entomol..

[11] Bailly-Choumara H, Kremer M (1970). Deuxième contribution à l’étude des *Culicoides* du Maroc (Diptera: Ceratopogonidae). Entomol Méd Parasitol..

[12] Kremer M, Hommel M, Bailly-Choumara H (1971). Troisième contribution à l’étude faunistique des *Culicoides* du Maroc. Ann Parasitol Hum Comp..

[13] Kremer M, Delécolle JC, Bailly-Choumara H, Chaker E (1979). Cinquième contribution à l’étude faunistique des *Culicoides* (Diptera, Ceratopogonidae). Entom Méd Parasitol..

[14] Chaker E, Bailly-Choumara H, Kremer M (1979). Sixième contribution à l’étude faunistique des Culicoides du Maroc (Diptera, Cefratopogonidae). Bull Inst Sci Rabat..

[15] Baylis M, Hasnaoui HE, Bouayoune H, Touti J, Mellor PS (1997). The spatial and seasonal distribution of African horse sickness and its potential *Culicoides* vectors in Morocco. Med Vet Ent..

[16] Bouayoune H, Touti J, El Hasnaoui H, Baylis M, Mellor PS (1998). The *Culicoides* vectors of African horse sickness virus in Morocco: distribution and epidemiological implications. Arch Virol..

[17] Chaker E, Sfari M, Rabhi M, Rouis M, Babba H, Azaiez R (2005). Note faunistique sur les *Culicoides* (Diptera, Ceratopogonidae) du Gouvernorat de Monastir (Tunisie). Parasite..

[18] Slama D, Chaker E, Mathieu B, Babba H, Depaquit J, Augot D (2014). Biting midges monitoring (Diptera: Ceratopogonidae: *Culicoides* Latreille) in the governate of Monastir (Tunisia): species composition and molecular investigations. Parasitol Res..

[19] Bourquia M, Garros C, Rakotoarivony I, Gardès L, Huber K, Boukhari I (2019). Update of the species checklist of *Culicoides* Latreille, 1809 biting midges (Diptera: Ceratopogonidae) of Morocco. Parasit Vectors..

[20] Sghaier S, Hammami S, Goffredo M, Hammami M, Portanti O, Lorusso A (2017). New species of the genus *Culicoides* (Diptera: Ceratopogonidae) for Tunisia, with detection of bluetongue viruses in vectors. Vet Ital..

[21] Kieffer J (1921). Sur quelques Diptères piqueurs de la Tribu Ceratopogoninae. Arch Inst Pasteur Afr Nord..

[22] Kieffer J (1922). Nouveaux Chironomides piqueurs habitant l’Algérie. Arch Inst Pasteur Alger..

[23] Kieffer J (1923). Ceratopogonines recueillis au Sahara Constantinois. Arch Inst Pasteur Alger..

[24] Goetghebuer M (1939). Cératopogonides et Chironomides recueillis en Algérie. Bull Ann Soc R Belge Ent..

[25] Clastrier J. Notes sur les Cératopogonidés. II. Quelques *Culicoides* d’Algérie à ailes tachetées. Arch Inst Pasteur Alger. 1957;35:404–44. 13522257

[26] Clastrier J. Notes sur les Cératopogonidés. III. *Culicoides semimaculatus* n. sp. d’Algérie. Arch Inst Pasteur Alger. 1958;36:55–60. 13534799

[27] Callot J, Kremer M, Basset M. *Culicoides marcleti* n. sp. et nouvelles localisations de culicoïdes (Diptères, Cératopogonidés) de la région méditerranéenne et particulièrement d’Algérie. Bull Soc Pathol Exot. 1968;61:271–82. 5755775

[28] Szadziewski R (1984). Ceratopogonidae (Diptera) from Algeria. VI. Culicoides Latr. Pol Pismo Entomol..

[29] Mellor PS, Hamblin C (2004). African horse sickness. Vet Res..

[30] Goffredo M, Catalani M, Federici V, Portanti O, Marini V, Mancini G (2015). Vector species of *Culicoides* midges implicated in the 2012–2014 bluetongue epidemics in Italy. Vet Ital..

[31] Goffredo M, Meiswinkel R, Federici V, Di Nicola F, Mancini G, Ippoliti C (2016). The ‘*Culicoides obsoletus* group’ in Italy: relative abundance, geographic range, and role as vector for bluetongue virus. Vet Ital..

[32] Paslaru AL, Mathis A, Torgerson P, Veronesi E (2018). Vector competence of pre-alpine *Culicoides* (Diptera: Ceratopogonidae) for bluetongue virus serotypes 1, 4 and 8. Parasit Vectors..

[33] Foxi C, Meloni G, Puggioni G, Manunta D, Rocchigiani A, Vento L (2019). Bluetongue virus detection in new *Culicoides* species in Sardinia. Italy. Vet Rec..

[34] Cetre-Sossah C, Madani H, Sailleau C, Nomikou K, Sadaoui H, Zientara S (2011). Molecular epidemiology of bluetongue virus serotype 1 isolated in 2006 from Algeria. Res Vet Sci..

[35] Kardjadj M (2017). An epidemiological overview of small ruminant diseases in Algeria. Rev Sci Tech OIE..

[36] Baldet T, Albina E, Delécolle JC (2003). Appui à la surveillance de la fièvre catarrhale ovine en Algérie.

[37] Baldet T, Delécolle JC (2006). Mission entomologique d’appui à la surveillance de la fièvre catarrhale ovine en Algérie.

[38] Nolan DV, Dallas JF, Piertney SB, Mordue (Luntz) AJ. Incursion and range expansion in the bluetongue vector *Culicoides imicola* in the Mediterranean basin: a phylogeographic analysis. Med Vet Entomol. 2008;22:340–51. 10.1111/j.1365-2915.2008.00744.x 19120962

[39] Djerbal M, Delécolle JC (2009). Entomological surveillance of bluetongue in Algeria. Rev Élev Méd Vét Pays Trop..

[40] Berrayah H, Hwang HS, Lee KY (2020). Molecular identification of *Culicoides* (Diptera: Ceratopogonidae) species in Algeria. Acta Trop..

[41] Belkharchouche M. Contribution à l’étude de la biodiversité des *Culicoides* (Diptera, Ceratopogonidae) responsable de la fièvre catarrhale dans la région Est-algerien foyer d’Oum-El-Bouaghi. MSc Thesis, Université d’Oum El Bouaghi, Algeria; 2014.

[42] Kabbout N. Contribution à l’étude bio écologique des insectes d’intérêt médical dans le Nord-Est Algérien. PhD thesis, Université d’Oum El Bouaghi, Algeria; 2017.

[43] Meddour R. Bioclimatologie, phytogéographie et phytosociologie en Algérie. Exemple des groupements forestiers et préforestiers de la Kabylie djurdjuréenne. PhD thesis, Université de Tizi Ouzou, Algeria; 2010.

[44] Wirth WW, Marston N (1968). A method for mounting small insects on microscope slides in Canada balsam. Ann Entomol Soc Am..

[45] Ramilo D, Garros C, Mathieu B, Benedet C, Allène X, Silva E, et al. Description of *Culicoides paradoxalis* sp. nov. from France and Portugal (Diptera: Ceratopogonidae). Zootaxa. 2013;3745:243. 10.11646/zootaxa.3745.2.4 25113346

[46] Szadziewski R, Filatov S, Dominiak P (2016). A redescription of *Culicoides griseidorsum* Kieffer, 1918, with comments on subgeneric position of some European taxa (Diptera: Ceratopogonidae). Zootaxa..

[47] Szadziewski R, Borkent A, Dominiak P. Fauna Europaea: Ceratopogonidae. 2001. In: Beuk P, Pape T, de Jong YSDM. Fauna Europaea: Diptera, Nematocera. Fauna Europaea version 2.6.2. 2013. https://www.fauna-eu.org. Accessed 3 June 2020.

[48] Gutsevich AV (1964). Biting midges of the genus *Culicoides* (Diptera, Heleidae) of the Ukrainian Carpathians (Transcarpathian province). Entomol Rev..

[49] Schwenkenbecher JM, Mordue AJ, Piertney SB (2009). Phylogenetic analysis indicates that *Culicoides dewulfi* should not be considered part of the *Culicoides obsoletus* complex. Bull Entomol Res..

[50] Lane RP (1983). Insects of Saudi Arabia *Culicoides* (Diptera: Ceratopogonidae) of Saudi Arabia and their potential veterinary importance. Fauna Saudi Arabia..

[51] Boorman J (1989). *Culicoides* (Diptera, Ceratopogonidae) of Arabian Peninsula with notes on their medical and veterinary importance. Fauna Saudi Arabia..

[52] Kremer M, Delécolle JC, Braverman Y (1991). A new and a redescription species of *Culicoides* from Sinai (Diptera, Ceratopogonidae). J Zool..

[53] Remm H, Zhogolev DT (1968). Contributions to the fauna of biting midges (Diptera, Ceratopogonidae) of the Crimea. Entomol Obozr..

[54] Glukhova VM (2005). *Culicoides* (Diptera, Ceratopogonidae) of Russia and adjacent lands. Int J Dipterol Res..

[55] Nielsen OB (1963). The biting midges of Lyngby Aamose (*Culicoides*: Ceratopogonidae).

[56] Gomulski LM, Meiswinkel R, Delécolle JC, Goffredo M, Gasperi G (2006). Phylogeny of the subgenus *Culicoides* and related species in Italy, inferred from internal transcribed spacer 2 ribosomal DNA sequences. Med Vet Entomol..

[57] Meiswinkel R, Gomulski LM, Delécolle JC, Goffredo M, Gasperi G (2004). The taxonomy of *Culicoides* vector complexes - unfinished business. Vet Ital..

[58] Nielsen SA, Kristensen M (2015). Delineation of *Culicoides* species by morphology and barcode exemplified by three new species of the subgenus *Culicoides* (Diptera: Ceratopogonidae) from Scandinavia. Parasit Vectors..

[59] Sarvašová A, Kočišová A, Candolfi E, Mathieu B. Description of *Culicoides bysta* n. sp., a new member of the Pulicaris group (Diptera: Ceratopogonidae) from Slovakia. Parasit Vectors. 2017;10:279. 10.1186/s13071-017-2195-4 PMC5457568 28578677

[60] Talavera S, Muñoz-Muñoz F, Verdún M, Pagès Martinez N. Morphology and DNA barcoding reveal three species in one: description of *Culicoides cryptipulicaris* sp. nov. and *Culicoides quasipulicaris* sp. nov. in the subgenus *Culicoides*. Med Vet Entomol. 2017;31:178–91. 10.1111/mve.12228 28370147

[61] Boorman J (1974). *Culicoides* (Diptera, Ceratopogonidae) from Cyprus. Cah ORSTOM..

[62] Delécolle JC. Deux *Culicoides* nouveaux pour la faune de France: *C. dendriticus* Boorman et *C. shaklawensis* Khalaf (Diptera, Ceratopogonidae). Bull Ass Philomathique Alsace Lorraine. 1995;31:37–46.

[63] Pudar D, Petrić D, Allène X, Alten B, Ayhan N, Cvetkovikj A (2018). An update of the *Culicoides* (Diptera: Ceratopogonidae) checklist for the Balkans. Parasit Vectors..

[64] Shevchenko AK (1967). Bloodsucking midges of the genus *Culicoides* (Diptera, Ceratopogonidae) from the valley of the middle current of the Desna. Entomol Obozr..

[65] Bourquia M. Déterminants environnementaux de la distribution des *Culicoides* (Diptera : Ceratopogonidae), moucherons vecteurs de virus animaux d’intérêt économique, au Maroc. Ph.D. thesis, IAV Hassan II, Morocco and Montpellier SupAgro, France; 2019.

[66] Kremer M, Deduit Y. Sur quelques *Culicoides* (Diptera: Ceratopogonidae) de Normandie. Description de *Culicoides picturatus* n. sp. Ann Parasitol Hum Comp. 1961;36:701–5. 10.1051/parasite/1961364700 14459566

[67] Sarvašová A, Goffredo M, Sopoliga I, Savini G, Kočišová A (2014). *Culicoides* midges (Diptera: Ceratopogonidae) as vectors of orbiviruses in Slovakia. Vet Ital..

[68] Du Toit RM (1944). The transmission of blue-tongue and horse-sickness by *Culicoides*. Onderstepoort J Vet Sci Anim Ind..

[69] Mellor PS, Boorman J, Baylis M (2000). *Culicoides* biting midges: their role as arbovirus vectors. Annu Rev Entomol..

[70] Balenghien T, Pagès N, Goffredo M, Carpenter S, Augot D, Jacquier E (2014). The emergence of Schmallenberg virus across *Culicoides* communities and ecosystems in Europe. Prev Vet Med..

[71] Ségard A, Gardes L, Jacquier E, Grillet C, Mathieu B, Rakotoarivony I (2017). Schmallenberg virus in *Culicoides* Latreille (Diptera: Ceratopogonidae) populations in France during 2011–2012 outbreak. Transbound Emerg Dis..

[72] Mellor PS, Pitzolis G (1979). Observations on breeding sites and light-trap collections of *Culicoides* during an outbreak of bluetongue in Cyprus. Bull Entomol Res..

[73] Savini G, Goffredo M, Monaco F, Di Gennaro A, De Santis P, Meiswinkel R (2004). The isolation of bluetongue virus from field populations of the Obsoletus Complex in central Italy. Vet Ital..

[74] De Liberato C, Scavia G, Lorenzetti R, Scaramozzino P, Amaddeo D, Cardeti G (2005). Identification of *Culicoides obsoletus* (Diptera: Ceratopogonidae) as a vector of bluetongue virus in central Italy. Vet Rec..

[75] Meiswinkel R, Van Rijn P, Leijs P, Goffredo M (2007). Potential new *Culicoides* vector of bluetongue virus in northern Europe. Vet Rec..

[76] Dijkstra E, van der Ven IJK, Meiswinkel R, Holzel DR, van Rijn PA, Meiswinkel R (2008). *Culicoides chiopterus* as a potential vector of bluetongue virus in Europe. Vet Rec..

[77] Venail R, Balenghien T, Guis H, Tran A, Setier-Rio ML, Delécolle JC, Mehlhorn H (2012). Assessing diversity and abundance of vector populations at a national scale: example of *Culicoides* surveillance in France after bluetongue virus emergence. Arthropods as vectors of emerging diseases.

[78] Romon P, Higuera M, Delécolle JC, Baldet T, Aduriz G, Goldarazena A (2012). Phenology and attraction of potential *Culicoides* vectors of bluetongue virus in Basque Country (northern Spain). Vet Parasitol..

[79] Foxi C, Delrio G, Falchi G, Marche MG, Satta G, Ruiu L (2016). Role of different *Culicoides* vectors (Diptera: Ceratopogonidae) in bluetongue virus transmission and overwintering in Sardinia (Italy). Parasit Vectors..

[80] Elbers ARW, Meiswinkel R, van Weezep E, van der Poel WHM, Kooi EA. Schmallenberg virus in *Culicoides* spp. biting midges, the Netherlands, 2011. Emerg Infect Dis. 2013;19:04. 10.3201/eid1901.121054 PMC3558002 23260040

[81] Goffredo M, Monaco F, Capelli G, Quaglia M, Federici V, Catalani M (2013). Schmallenberg virus in Italy: a retrospective survey in *Culicoides* stored during the bluetongue Italian surveillance program. Prev Vet Med..

[82] Larska M, Lechowski L, Grochowska M, Żmudziński JF. Detection of the Schmallenberg virus in nulliparous *Culicoides obsoletus/scoticus* complex and *C. punctatus*, the possibility of transovarial virus transmission in the midge population and of a new vector. Vet Microbiol. 2013;166:467–73. 10.1016/j.vetmic.2013.07.015 23928121

[83] De Regge N, Deblauwe I, De Deken R, Vantieghem P, Madder M, Geysen D, et al. Detection of Schmallenberg virus in different *Culicoides* spp. by real-time RT-PCR. Transbound Emerg Dis. 2012;59:471–5. 10.1111/tbed.12000 23025501

[84] Rasmussen LD, Kristensen B, Kirkeby C, Rasmussen TB, Belsham GJ, Bødker R (2012). *Culicoides* as vectors of Schmallenberg virus. Emerg Infect Dis..

[85] Elbers ARW, Meiswinkel R, van Weezep E, Kooi EA, van der Poel WHM (2015). Schmallenberg virus in *Culicoides* biting midges in the Netherlands in 2012. Transbound Emerg Dis..

[86] Carpenter S, McArthur C, Selby R, Ward R, Nolan DV, Luntz AM (2008). Experimental infection studies of UK *Culicoides* species midges with bluetongue virus serotypes 8 and 9. Vet Rec..

[87] Caracappa S, Torina A, Guercio A, Vitale F, Calabro A, Purpari G (2003). Identification of a novel bluetongue virus vector species of *Culicoides* in Sicily. Vet Rec..

[88] Hilali M, Abu-Elzein ET, Al-Afaleq A, Boorman J, Alatyia S, Alnaeem A (2003). *Culicoides* midges (Ceratopogonidae) in some localities of Saudi Arabia and their veterinary significance. Vet Arh..

[89] Alahmed AM, Kheir SM, Al Khereiji MA (2010). Distribution of *Culicoides* Latreille (Diptera: Ceratopogonidae) in Saudi Arabia. J Entomol..

[90] El Sinnary K, Hussein HS (1980). *Culicoides kingi*, Austen: a vector of *Onchocerca gutturosa* (Neumann, 1910) in the Sudan. Ann Trop Med Parasitol..

[91] Bakhoum MT, Sarr M, Fall AG, Huber K, Fall M, Sembène M (2018). DNA barcoding and molecular identification of field-collected *Culicoides* larvae in the Niayes area of Senegal. Parasit Vectors..

[92] Lee VH (1979). Isolation of viruses from field populations of *Culicoides* (Diptera: Ceratopogonidae) in Nigeria. J Med Entomol..

[93] Mellor PS, Osborne R, Jennings DM. Isolation of bluetongue and related viruses from *Culicoides* spp. in the Sudan. Epidemiol Infect. 1984;93:621–8. 10.1017/s0022172400065190 PMC2129472 6096444

