# Correction to: Update of the *Culicoides* (Diptera: Ceratopogonidae) species checklist from Algeria with 10 new records

**DOI:** 10.1186/s13071-021-04825-z

**Published:** 2021-07-12

**Authors:** Mounira Belkharchouche, Selima Berchi, Bruno Mathieu, Ignace Rakotoarivony, Maxime Duhayon, Thierry Baldet, Thomas Balenghien

**Affiliations:** 1 Ecole Nationale Supérieure de Biotechnologie, Taoufk Khaznadar, nouveau pôle universitaire Ali Mendjeli, B.P. E66, 25100, Constantine, Algérie; 2 Faculté des Sciences de la Nature et de la Vie, Université Ibn Khaldoun, B.P.75 Zaaroura, Tiaret, 1400 Algérie; 3 Laboratoire de Biosystématique et Ecologie des Arthropodes, Faculté des Sciences de la Nature et de la Vie, Département de Biologie Animale, Université Frères Mentouri, Constantine, 1 2500 Algérie; 4 CIRAD, UMR ASTRE, 34398 Montpellier, France; 5 Institut de Parasitologie et de Pathologies Tropicales de Strasbourg (IPPTS), UR 7292, 3 Rue Koeberlé, 67000 Strasbourg, France; 6 ASTRE, University of Montpellier, CIRAD, INRAE, Montpellier, France; 7 CIRAD, UMR ASTRE, Sainte-Clotilde, 97491 La Réunion, France; 8 CIRAD, UMR ASTRE, 10101 Rabat, Morocco; 9 Institut Agronomique et Vétérinaire Hassan II, Unité Microbiologie, Immunologie et Maladies Contagieuses, 10100 Rabat, Morocco

## Correction to: Parasites Vectors (2020) 13: 463https://doi.org/10.1186/s13071-020-04335-4

Following publication of the original article [1], it was brought to the authors’ attention that Table 1 had published with incomplete information.

**Table 1:** List of the material examined to produce 10 new records for the Algerian fauna

Species	Type of material	Country	Locality	Coordinates	Date	Material examined
*Culicoides* (*Avaritia*) *chiopterus* (Meigen)	Non-type material	Algeria	Ain El Deheb	34°51'N, 1°29'E	23–24 Mar 2018	1 female
		France	Andevanne	49°23'N, 5°4'E	17–18 Oct 2006	1 female
			Caro	43°8'N, 1°14'W	23–24 Oct 2006	1 female
			Nuillé sur Vicoin	47°57'N, 0°45'W	4–5 Jul 2011	1 female
			Orbey	48°7'N, 7°7'E	25–26 May 2009	5 females
			Pontivy	48°4'N, 2°58'W	18–19 Jan 2012	5 females
			Schwobsheim	48°13'N, 7°34'E	18–19 Apr 2011	1 female
*Culicoides* (*Avaritia*) *dewulfi* Goetghebuer	Non-type material	Algeria	Tiaret	35°23'N, 1°21'E	16–19 Sep 2017	1 female
			Bougara	35°29'N, 1°55'E	13–15 Nov 2016	1 female
			Sougueur	35°09'N, 1°31'E	13–16 May 2016	2 females
			Ain El Deheb	34°51'N, 1°29'E	25–27 Jul 2017	3 females
			Ain El Deheb	34°51'N, 1°29'E	1–2 Jun 2018	1 female
			Hammadia	35°26'N, 1°53'E	2–5 Jun 2016	1 female
			Takhmaret	35°06'N, 0°42'E	12–15 Sep 2017	1 female
			Takhmaret	35°06'N, 0°42'E	13–14 Sep 2018	3 females
		France	Caro	43°84 N, 1°14'W	29–30 May 2006	2 females
			Cuguen	48°26'N, 1°39'W	14–15 Nov 2011	1 female
			Lazer	44°21'N, 5°51'E	18–19 Apr 2011	2 females
*Culicoides* (*Beltranmyia*) *navaiae* Lane	Non-type material	Algeria	Tiaret	35°23'N, 1°21'E	3–6 Jun 2017	2 females
			Tiaret	35°23'N, 1°21'E	28–30 Sep 2017	1 male, 2 females
			Tiaret	35°23'N, 1°21'E	21–22 Sep 2018	2 females
			Bougara	35°29'N, 1°55'E	9–12 May 2016	3 females
			Bougara	35°29'N, 1°55'E	10–13 Nov 2016	3 females
			Bougara	35°29'N, 1°55'E	15–19 Apr 2017	5 females
			Bougara	35°29'N, 1°55'E	29–30 Jun 2018	1 female
			Sougueur	35°09'N, 1°31'E	3–6 Feb 2016	11 females
			Sougueur	35°09'N, 1°31'E	23–26 Jun 2017	4 females
			Sougueur	35°09'N, 1°31'E	14–15 Sep 2018	2 females
			Ain El Deheb	34°51'N, 1°29'E	2–5 Apr 2016	11 females
			Ain El Deheb	34°51'N, 1°29'E	24–27 Apr 2017	2 females
			Ain El Deheb	34°51'N, 1°29'E	21–22 Sep 2018	27 females
			Ksar Chellala	35°15'N, 2°18'E	13–16 May 2016	20 females
			Ksar Chellala	35°15'N, 2°18'E	1–4 Nov 2017	18 females
			Ksar Chellala	35°15'N, 2°18'E	21–22 Sep 2018	18 females
			Hammadia	35°26'N, 1°53'E	16–19 Jun 2016	12 females
			Hammadia	35°26'N, 1°53'E	1–4 Jul 2017	18 females
			Hammadia	35°26'N, 1°53'E	7–8 Sep 2018	9 females
			Rahouia	35°29'N, 1°03'E	23–26 Jun 2017	22 females
			Rahouia	35°29'N, 1°03'E	9–12 Jul 2017	11 females
			Rahouia	35°29'N, 1°03'E	21–22 Sep 2018	18 females
			Machraa S’fa	35°22'N, 1°03'E	3–6 Jun 2017	12 females
			Machraa S’fa	35°22'N, 1°03'E	23–26 Jun 2017	23 females
			Machraa S’fa	35°22'N, 1°03'E	13–14 Jul 2018	7 females
			Takhmaret	35°06'N, 0°42'E	5–8 Jul 2016	8 females
			Takhmaret	35°06'N, 0°42'E	29 Jul–1 Aug 2017	6 females
			Takhmaret	35°06'N, 0°42'E	8–9 Jun 2018	9 females
		Egypt	Sinaï, Khirba	31°1'N, 32°53'E	25 Apr 1979	10 females
			Sinaï, Khirba	31°1'N, 32°53'E	24 Apr 1979	1 male
*Culicoides* (*Culicoides*) *grisescens* Edwards	Non-type material	Algeria	Sougueur	35°09'N, 1°31'E	5–7 Apr 2017	10 females
			Sougueur	35°09'N, 1°31'E	26–29 May 2017	13 females
			Sougueur	35°09'N, 1°31'E	3–6 Jun 2017	5 females
			Sougueur	35°09'N, 1°31'E	17–20 Jul 2017	13 females
			Sougueur	35°09'N, 1°31'E	25–28 Jul 2017	11 females
			Sougueur	35°09'N, 1°31'E	20–23 Sep 2017	15 females
			Sougueur	35°09'N, 1°31'E	1–3 Oct 2017	12 females
			Sougueur	35°09'N, 1°31'E	1–4 Jul 2018	9 females
			Sougueur	35°09'N, 1°31'E	7–8 Sep 2018	9 females
			Ain El Deheb	34°51'N, 1°29'E	29 Nov –5 Dec 2015	2 females
			Ain El Deheb	34°51'N, 1°29'E	15–17 Sep 2016	15 females
			Ain El Deheb	34°51'N, 1°29'E	5–7 Apr 2017	9 females
			Ain El Deheb	34°51'N, 1°29'E	28–29 Sep 2018	11 females
			Ksar Chellala	35°15'N, 2°18'E	26–29 May 2017	7 females
			Ksar Chellala	35°15'N, 2°18'E	9–12 Nov 2017	6 females
			Ksar Chellala	35°15'N, 2°18'E	21–22 Sep 2018	16 females
			Hammadia	35°26'N, 1°53'E	15–17 Sep 2016	8 females
			Hammadia	35°26'N, 1°53'E	28–30 Sep 2017	7 females
			Hammadia	35°26'N, 1°53'E	1–3 Oct 2017	11 females
			Hammadia	35°26'N, 1°53'E	28–29 Sep 2018	12 females
			Rahouia	35°29'N, 1°03'E	24–27 Jun 2016	5 females
			Rahouia	35°29'N, 1°03'E	12–15 Sep 2017	8 females
			Rahouia	35°29'N, 1°03'E	21–22 Sep 2018	7 females
			Machraa S’fa	35°22'N, 1°03'E	8–10 Sep 2016	9 females
			Machraa S’fa	35°22'N, 1°03'E	26–29 May 2017	9 females
			Machraa S’fa	35°22'N, 1°03'E	15–16 Jun 2018	7 females
			Takhmaret	35°06'N, 0°42'E	20–23 Jun 2016	19 females
			Takhmaret	35°06'N, 0°42'E	5–7 Apr 2017	10 females
			Takhmaret	35°06'N, 0°42'E	28–29 Sep 2018	12 females
		France	Brognon	49°55'N, 4°18'E	2è–28 Jun 2007	1 female
			Marcoux	44°7'N, 6°17'E	22–23 Jul 2009	4 females
*Culicoides* (*Culicoides*) *paradoxalis* Ramilo & Delécolle	Type material	Algeria	Ain El Deheb	34°51'N, 1°29'E	27–28 Apr 2018	1 female
		France, Corsica	Pietra Corbara	42°50'N, 9°26'E	3–4 Jun 2003	1 female (holotype)
			Pietra Corbara	42°50'N, 9°26'E	3–4 Jun 2003	2 female
			Pietra Corbara	42°50'N, 9°26'E	6–7 Jul 2004	1 female
			Porto Vecchio	41°35'N, 9°15'E	22–23 Sep 2005	2 females
			Sarrola Carcopino	42°0'N, 8°50'E	26–27 Jun 2006	1 female
			Sartene	41°38'N, 8°57'E	27–28 Sep 2005	1 female
			Sartene	41°38'N, 8°57'E	12–13 Jun 2002	1 female
			Sartene	41°38'N, 8°57'E	20–21 Jun 2002	1 female
*Culicoides* (*Sensiculicoides*) *shaklawensis* Khalaf	Non-type material	Algeria	Tiaret	35°23'N, 1°21'E	9–14 Mar 2016	1 female
			Tiaret	35°23'N, 1°21'E	15–16 Jun 2018	1 female
			Tiaret	35°23'N, 1°21'E	27–28 Jul 2018	1 female
			Bougara	35°29'N, 1°55'E	29–31 Oct 2016	1 female
		France	Calvi	42°32'N, 8°45'E	23–24 Jun 2003	1 female
			Marcoux	44°7'N, 6°17'E	22–23 Jul 2009	1 female
			Moltifao	42°28'N, 9°7'E	5–6 Aug 2003	1 female
*Culicoides* (*Sensiculicoides*) *simulator* Edwards	Non-type material	Algeria	Tiaret	35°23'N, 1°21'E	1–2 Jun 2018	1 female
		France	Aleria	42°6'N, 9°29'E	8–9 Jun 2004	1 female
			La Chapelle d’Andaine	48°31'N, 0°28'W	19–20 Jun 2012	1 female
			Marcoux	44°7'N, 6°17'E	22–23 Jul 2009	1 female
			Remilly Aillicourt	49°39'N, 4°58'E	24–25 May 2007	1 female
*Culicoides* (*Sensiculicoides*) *univittatus* Vimmer	Non-type material	Algeria	Tiaret	35°23'N, 1°21'E	10–12 Dec 2016	1 male, 1 female
			Tiaret	35°23'N, 1°21'E	15–17 Mar 2017	4 males, 7 females
			Tiaret	35°23'N, 1°21'E	4–5 May 2018	2 females
		France, Corsica	Figari	41°30'N, 9°5'E	10–11 Apr 2006	1 male, 4 females
			Figari	41°30'N, 9°5'E	14–15 Feb 2011	1 female
			Moltifao	42°28'N, 9°7'E	25–26 Apr 2005	1 female
			San Giuliano	42°17'N, 9°32'E	6–7 Mar 2003	2 males
			San Giuliano	42°17'N, 9°32'E	10–11 Feb 2011	1 female
*Culicoides* (*Silvaticulicoides*) *achrayi* Kettle & Lawson	Non-type material	Algeria	Hammadia	35°26'N, 1°53'E	27–28 Apr 2018	1 female
			Sougueur	35°09'N, 1°31'E	15–17 Oct 2016	1 female
			Sougueur	35°09'N, 1°31'E	5–7 Apr 2017	1 female
		France	Crozon	48°16'N, 4°30'W	13–14 Jun 2011	2 females
			Pontivy	48°4'N, 2°58'W	18–19 Jan 2012	3 females
*Culicoides* (*Silvaticulicoides*) *picturatus* Kremer & Deduit	Non-type material	Algeria	Tiaret	35°23'N, 1°21'E	24–27 Jun 2016	1 female, 1 male
			Tiaret	35°23'N, 1°21'E	10–12 Jul 2016	1 female
			Tiaret	35°23'N, 1°21'E	28–29 Sep 2018	1 female
		France	Humes Jorquenay	47°54'N, 5°18'E	6–7 Jun 2011	1 male
			Lazer	44°21'N, 5°51'E	18–19 Apr 2011	1 female
			Porto Vecchio	41°35'N, 9°15'E	29–30 May 2002	2 males
			Saignon	43°51'N, 5°27'E	6–7 Jun 2011	3 females

The published article has since been updated and the corrected table may be found in this correction.

The authors apologize for any inconvenience caused.
